# Mating-Induced Transcriptome Changes in the Reproductive Tract of Female *Aedes aegypti*

**DOI:** 10.1371/journal.pntd.0004451

**Published:** 2016-02-22

**Authors:** Catalina Alfonso-Parra, Yasir H. Ahmed-Braimah, Ethan C. Degner, Frank W. Avila, Susan M. Villarreal, Jeffrey A. Pleiss, Mariana F. Wolfner, Laura C. Harrington

**Affiliations:** 1 Department of Entomology, Cornell University, Ithaca, New York, United States of America; 2 Instituto Colombiano de Medicina Tropical - Universidad CES, Medellín, Colombia; 3 Department of Biology, University of Rochester, Rochester, New York, United States of America; 4 Department of Molecular Biology and Genetics, Cornell University, Ithaca, New York, United States of America; University of Perugia, ITALY

## Abstract

The *Aedes aegypti* mosquito is a significant public health threat, as it is the main vector of dengue and chikungunya viruses. Disease control efforts could be enhanced through reproductive manipulation of these vectors. Previous work has revealed a relationship between male seminal fluid proteins transferred to females during mating and female post-mating physiology and behavior. To better understand this interplay, we used short-read RNA sequencing to identify gene expression changes in the lower reproductive tract of females in response to mating. We characterized mRNA expression in virgin and mated females at 0, 6 and 24 hours post-mating (hpm) and identified 364 differentially abundant transcripts between mating status groups. Surprisingly, 60 transcripts were more abundant at 0hpm compared to virgin females, suggesting transfer from males. Twenty of these encode known *Ae. aegypti* seminal fluid proteins. Transfer and detection of male accessory gland-derived mRNA in females at 0hpm was confirmed by measurement of eGFP mRNA in females mated to eGFP-expressing males. In addition, 150 transcripts were up-regulated at 6hpm and 24hpm, while 130 transcripts were down-regulated at 6hpm and 24hpm. Gene Ontology (GO) enrichment analysis revealed that proteases, a protein class broadly known to play important roles in reproduction, were among the most enriched protein classes. RNAs associated with immune system and antimicrobial function were also up-regulated at 24hpm. Collectively, our results suggest that copulation initiates broad transcriptome changes across the mosquito female reproductive tract, “priming” her for important subsequent processes of blood feeding, egg development and immune defense. Our transcriptome analysis provides a vital foundation for future studies of the consequences of mating on female biology and will aid studies seeking to identify specific gene families, molecules and pathways that support key reproductive processes in the female mosquito.

## Author Summary

Female post-mating behavior has important consequences for mosquito populations and their ability to transmit diseases. Male *Aedes aegypti* seminal fluid substances transferred during mating cause many important changes to female behavior and physiology, including blood feeding behavior, egg development, and oviposition. In an effort to understand how males induce these responses in *Ae. aegypti* females, we characterized the transcriptome changes that occur in the female reproductive tract at different time points after mating. We found several RNAs that are apparently transferred by the male, and 280 genes whose mRNA abundance in the female is affected by mating. The nature of the predicted products of many of these genes suggests roles in priming the reproductive tract for egg development, protecting the female against bacterial infections or processing the blood meal. This identification of mating-responsive genes provides information potentially useful for developing tools aimed at preventing disease transmission by manipulating female mosquitoes’ post-mating responses.

## Introduction

*Aedes aegypti* is a major vector of arboviruses that impact human health, including those causing dengue and chikungunya [1]. Collectively, these neglected infections cause significant human morbidity and mortality. Among these arboviral threats, dengue is considered the most important, as it affects an estimated 390 million people worldwide per year, with 500,000 episodes of severe dengue and >20,000 dengue related deaths [2, 3]. In addition, chikungunya is sweeping across the globe, with nearly 1 million cases in the Americas in 2015 alone [4]. With no effective antiviral therapy, no commercially licensed vaccine, and no treatment for dengue or chikungunya, control efforts for these diseases must focus on developing tools for vector control that will ultimately reduce the burden of human infections [5]. Tools that target key processes in *Ae. aegypti* reproduction hold significant promise.

In recent years, progress has been made towards understanding the *Ae. aegypti* mating system. This work builds upon early observations that, after mating, *Ae. aegypti* females undergo profound physiological and behavioral changes, including increased egg development and oviposition rates [6, 7], changes in host-seeking and feeding behavior [8], and increased mating refractoriness [9, 10]. These changes largely result from the receipt of seminal fluid proteins (Sfps) that are transferred from males to females along with sperm during copulation [9, 11, 12].

Given the dramatic changes in a female’s physiology and behavior after mating, it is likely that mating triggers large changes in gene expression, yet nothing is known about mating-induced changes in gene expression in *Ae. aegypti* females. However, mating-induced transcriptome changes in the female have been studied at the whole-body level in other insects, including *Drosophila melanogaster*[13–17], *Apis mellifera*[18], *Ceratitis capitata*[19], and *Anopheles gambiae*[20], as well as in the *D. melanogaster* and *An. gambiae* reproductive tract (RT) [21–23].

Gene expression profiles of *D. melanogaster* whole females revealed that, soon after copulation (1–3 hours post-mating; hpm hereafter), a large number of genes showed small-magnitude changes in transcript levels. In contrast, by 6hpm, larger magnitude changes were observed for a smaller number of genes [15]. In addition, *D. melanogaster* females that receive Sfps, but not sperm, show differential expression of 160 genes at 1–3 hpm. Some immunity-related genes, especially those encoding antimicrobial peptides, were strongly up-regulated in response to Sfp receipt. Other categories of genes whose transcript levels were modulated by the transfer of Sfps include ones that encode enzymes, receptors, and transporters, some of which are involved in metabolism [14].

Microarray analysis of the transcriptome of the *D. melanogaster* female lower RT (reproductive organs below the oviducts) revealed differential expression of 539 genes in virgin versus mated females at 0, 3, 6 or 24hpm [21]. Interestingly, there was a pronounced peak in the number of differentially expressed RNAs at 6hpm, a time when most Sfps are at low levels or are no longer detectable [24, 25]. Many of these mating-regulated genes encode proteins with predicted functions in catalytic activity and in nucleic acid binding. Microarray analyses of RNAs from the oviduct of mated *D. melanogaster* females identified 432 transcripts expressed differentially in mated relative to unmated females. Among these RNAs, those from immune response genes showed a substantial increase in expression [22]. In *An. gambiae*, differential expression was detected for RNAs encoding various proteases in whole mated females at 2, 6 and 24hpm relative to virgin females. Some of those RNAs were also up-regulated in females that had mated to spermless males [26], indicating that their abundance was regulated by mating or seminal molecules rather than by sperm (analogous to results previously obtained in *Drosophila*[14]). Microarray analysis of the *An. gambiae* lower RT at 3, 12, and 24hpm showed that there is significant upregulation of genes involved in metabolic processes, particularly at 24hpm [23], in agreement with studies of expression profiles of the spermathecae that show increased expression of metabolic genes after 24 hours [27].

A recent paper reported transcriptome profiles from the fat body of *Ae. aegypti* after blood-feeding [28]. Another recent paper described gene expression changes in reproductive tissues of males and females before and after blood feeding [29]. However, post-mating transcriptome changes have not been explored for the female RT after mating. Identification of specific genes and gene classes whose expression is regulated post-mating is vital to understanding the reproductive biology of this disease vector.

We are especially interested in exploring the link between mating and downstream reproductive processes in the *Ae. aegypti* natural mating system. For this mosquito, the host serves as an encounter site for mating [30]. Males tend to patrol the space around the human host and intercept females as they fly in to take a blood meal [31]. For this reason, the first mating (and often only mating for this primarily monandrous species [32]), blood ingestion, and the beginning of the first gonotrophic cycle are likely to occur sequentially. Given that mating has already been shown to accelerate oocyte development, we predicted that the male might thereby “prime” the entire RT for these subsequent reproductive stages.

Here, we report mating-induced changes in mRNA levels in the *Ae. aegypti* female RT. Since we were specifically interested in the male’s potential to prime the female reproductive system, we examined changes that occurred in the female RT without the ovaries as this is the primary site of interaction between the male ejaculate and the female. Using short read RNA sequencing (RNAseq), we compared transcript abundances in the RTs of virgin females to females at three time points after copulation (0, 6 and 24hpm). We chose the 6 and 24hpm time points to capture intervals before females typically produce their first egg batch (∼72 or more hours depending on ambient temperature [33]) and to compare with studies conducted in *Anopheles* and *Drosophila*. To consider the potential for transfer of male-derived Sfp transcripts to females, as was observed in *D. mojavensis*[34], we also included a 0hpm time point. We identified a total of 364 transcripts that show significantly different abundance levels between the RTs of virgin and mated females. A subset of these transcripts is likely male-derived, as their abundance increases dramatically immediately after mating. Furthermore, we find that several gene ontology (GO) categories are enriched among transcripts that are differentially regulated in response to mating, especially among up-regulated transcripts at 6hpm and 24hpm. We discuss these findings in the context of known reproductive processes in other insects and as potential targets for vector control.

## Materials and Methods

### Mosquito rearing

We used *Aedes aegypti* originally collected in Bangkok, Thailand (15.7193°N, 101.752°E) in 2011 and supplemented with field material from the same site in 2012 and 2014. Mosquitoes were reared as described in [35]. Individual pupae were transferred to vials to ensure virginity and sorted by sex upon eclosion. Two hundred individuals were transferred into 8L plastic cages, separated by sex, and held in single-sex groups until experiments commenced.

### Mosquito mating

Matings were conducted as described previously [10]. Briefly, 5 to 6 day-old virgin *Ae. aegypti* males and females were used for each mating. Each virgin female was released into an 8L observation cage containing approximately 8 virgin males. Male and female couples were observed carefully, and copulating pairs were removed using a mouth aspirator after a minimum mating duration of 8 seconds to ensure successful copulation [36, 37]. In addition, a subset of females were examined for sperm in the spermathecae or in the bursa during dissections. Mosquitoes from the same cohort were used for the virgin samples and the three post-mating time points. Females in the 0hpm treatment were placed on ice immediately after mating. Females from the later post-mating time points were individually transferred into 50mL test tubes after mating and held in an environmental chamber at 28 ± 1°C with 71.9 ± 9.5% relative humidity and a photoperiod of 10h light:10h dark (with a 2h simulated dusk and dawn period). At the 6 or 24hpm, females were placed on ice and immediately dissected.

To test for transfer of male-derived transcripts to females during copulation, virgin females were mated with transgenic males that express eGFP in the accessory glands, driven by the promoter of the Sfp, AAEL010824 [35]. Mated females were placed on ice and immediately dissected after copulation.

### Dissections

To dissect reproductive tracts (RTs), females were anesthetized on ice and placed on a chilled glass slide in a droplet of phosphate-buffered saline (Sigma, St Louis, MO). Dissections were carried out by first dissecting the 0hpm samples, followed by the virgin samples and the 6hpm samples. The 24hpm samples were dissected around the same time the following day. Tissues for each replicate were pooled from several days of dissections. The RT included the bursa, the three spermathecae, the common oviduct and the two lateral oviducts. Care was taken to remove any extraneous tissues, including the adherent fat body and ovaries. Immediately after dissection, RTs were placed in 150*μ*L of Trizol (Invitrogen, Carlsbad, California) and stored on ice. For RNAseq libraries, between 40 and 60 lower RTs were extracted to obtain ∼1*μ*g of total RNA for each replicate of the virgin and post-mating treatments (three replicates for each of the four samples). To test for eGFP mRNA transfer from males, RNA was extracted from RTs of 4 or 5 females mated with AAEL010824-eGFP males.

### RNA extraction, library preparation and read processing

Immediately after dissection, tissues were homogenized with a pestle, and an additional 350*μ*L Trizol was added to each sample. Samples were incubated at room temperature for 5 min and stored at -80°C. RNA was then prepared by chloroform/isopropanol extraction and ethanol precipitation following the manufacturer’s protocol (Life technologies, Grand Island, NY). The concentration of RNA in each sample was measured using a Qubit spectrophotometer (Invitrogen, Grand Island, NY), and the quality of RNA was measured on a Fragment Analyzer (Advanced Analytical Technologies, Inc., Ames, IA).

From total RNA, mRNA was extracted using the poly-A mRNA Magnetic Isolation Module (New England Biolabs, Inc., Ipswich, MA). cDNA libraries for each replicate were prepared using the NEBNext Ultra RNA Library Prep Kit for Illumina (New England Biolabs, Inc., Ipswich, MA). NEBNext Multiplex Oligos for Illumina (New England Biolabs, Inc., Ipswich, MA) were ligated to each library prior to sequencing.

Samples were sequenced on the Illumina platform (HiSeq 2500, Cornell University Biotechnology Resource Center) in two rounds. In the first, all samples were sequenced in one lane and the reads were analyzed for consistency among replicates (see below and S1 Methods). It was found that two of the virgin repicates and one 24hpm replicate contained a large number of reads derived from rRNAs; thus, these samples were considered to be contaminated with ribosomal RNA, and were not included in the final analyses. Rather, in the second round of analysis, one of the virgin replicates and two replicates of each of the mated samples were re-sequenced at higher depth and used in subsequent analyses in place of the rRNA-contaminated libraries.

Single-end, 100bp reads were processed by trimming the first 10 bases and end bases below a quality Phred score of 20 (FASTX-TOOLKIT v.0.0.13). In addition, reads having an average quality score below 20 were discarded.

### Read alignment and differential expression analysis

To generate the transcriptome used for differential expression analysis, each sample was mapped individually to the *Ae. aegypti* genome (release AaegL2, VectorBase) (Tophat v.2.0.9). The *Ae. aegypti* annotation file (release AaegL2.2) was used as a guide for known transcripts, allowing us to circumvent the need to heavily annotate the transcriptome *de novo*. Mapped reads from each sample were assembled into transcript fragments (Cufflinks v.2.2.1), and assembly annotations from each sample were merged to produce the gene annotation file used to define transcripts for differential expression analysis. Spliced transcript sequences were then reconstructed from the genome sequence using the merged gene annotation file (Cufflinks “gffread” utility, v.2.2.1). We also analyzed the data for changes in splice-isoforms. The final transcriptome consisted of 23,770 genes, which comprised 42,858 isoforms.

To perform differential expression testing among samples, each replicate was individually mapped to the transcriptome (Bowtie2 v.2.2.2) and the raw number of reads mapping to each transcript was estimated (RSEM v.1.2.8). The read counts were processed and normalized using the Trimmed Mean of M-values (TMM) method to obtain reads per kilobase of transcript per million mapped reads (RPKM) values. A quality filter was established by estimating a minimum read count (180 reads/transcript) in any given condition, above which a 2-fold change in expression between virgin and post-mating samples would be considered reliable; 10,031 transcripts satisfy this minimum read count threshold (S1 Methods). Additionally, to assess consistency among replicates, the fold change between replicates of each sample was examined; a total of 40 transcripts showed higher than 2-fold difference between replicates of the same sample and were excluded from further analysis. For each transcript that passed this filter, the fold change, *p*-value and false discovery rate (FDR) were calculated separately for each of the virgin-0hpm, virgin-6hpm, and virgin-24hpm comparisons (edgeR v. 3.2.4). The virgin-0hpm comparison revealed a set of transcripts that are likely male-derived, and were thus analyzed separately from later time point comparisons (these potentially male-derived transcripts were removed from subsequent analyses). Differentially expressed transcripts with similar expression profiles from the virgin-6hpm and virgin-24hpm comparisons were grouped by *K*-means clustering (R “Cluster” package).

### Functional annotation of transcriptome sequences

An annotation database for the transcriptome produced here was created according to the Trinotate guidelines for transcriptome annotation (v. r20140708). First, transcriptome sequences were examined for the presence of coding sequences by *in silico* prediction of open reading frames (ORFs) with a minimum peptide length of 20 amino acids (Transdecoder r20140116). Second, homology to known transcripts was assessed by generating BLASTp and BLASTx reports against the SwissProt database (www.uniprot.org). Finally, homologous protein domains (hmmscan v.2.3.2), predicted signal peptides (signalP v.4.1), and predicted transmembrane domains (tmHMM v.2.0) were identified for each protein sequence. This comprehensive annotation database was used to query possible gene functions and to perform downstream Gene Ontology (GO) enrichment analysis. GO enrichment analyses were performed for the set of up- and down-regulated transcripts and for clusters with similar expression profiles (GOSeq:R package v.1.18.0; DAVID v.6.7).

### Quantitative real-time PCR (qRT-PCR)

To validate the expression profiles of differentially expressed transcripts identified through RNAseq, transcript abundance was measured at different time points for seven genes using qRT-PCR. Total RNA from RTs was isolated as described above (samples were independent from those used for RNAseq analysis). Prior to cDNA synthesis, RNA was treated with RNase-free DNAse (Clontech, Madison, Wisconsin). Reverse transcription was performed using 1*μ*g of total RNA and oligo dT following the manufacturer’s instructions (Clontech, Madison, Wisconsin). Relative transcript levels of each gene were measured using quantitative PCR conducted with a CFV96 Real-Time System (Bio-rad, Hercules, California). For primer sequences used, see S1 Table. Amplifications were carried out in a 15*μ*l reaction containing 7.5*μ*l of iQ SYBR Green Supermix (Bio-rad, USA), 1*μ*l cDNA and 300nM of each primer. Cycle differences between genes and *RpS17* and *Actin* (*ΔΔ*CT) were compared to generate the relative expression of the transcripts at different time points before and after mating. Two different housekeeping genes were included to ensure that mating did not influence transcript abundance of one of those genes. The fold-change in the RNAseq experiment and the qRT-PCR were compared using a Pearson correlation coefficient. In addition, qRT-PCR was used to test eGFP mRNA transfer from males to females during copulation, following the methods describe above. *ΔΔ*CT for eGFP transfer was generated using *RpS17* for normalization.

## Results and Discussion

*Aedes aegypti* undergoes drastic physiological and behavioral changes after mating that result from the receipt of seminal fluid. Because the reproductive tract plays a crucial role in seminal receipt, sperm storage and ejaculate processing, and, ultimately, in egg development and oviposition, we were interested in understanding gene expression changes specifically in RT tissues. To examine post-mating transcriptome changes within the female RT, we compared transcript abundance levels in virgin females with those in mated females at 6 and 24hpm. We chose the 6hpm time point based on results from *Drosophila*, in which the peak of post-mating expression (the number of differentially expressed RNAs and/or the magnitude of fold-change) was highest [15, 21]. We chose to examine 24hpm to allow comparison to *An. gambiae*, whose peak differential expression was observed at this time [20], allowing us to identify transcriptome responses persistent throughout Culicidae. Finally, because male-derived mRNAs have been reported to be transferred during mating in *D. mojavensis*[34], we tested whether this phenomenon occurs in *Ae. aegypti* by comparing the RT transcript abundances of virgin females to those extracted from females immediately after mating (0hpm).

### Post-mating transcriptome analysis of the reproductive tract of *Ae. aegypti* females

We measured abundance levels of 23,770 assembled transcripts (6,481 previously unannotated), 10,031 of which (1,143 previously unannotated) passed our quality control filter (described above and in S1 Methods). Significance tests for each gene that passed the filter were conducted for each of the virgin-0hpm, virgin-6hpm, and virgin-24hpm comparisons. We only considered transcripts that had a 2-fold or greater change in abundance between virgin and post-mating samples. In addition, a transcript was considered significantly differentially expressed if the significance test *p*-value was below 0.05 after correcting for multiple tests (Benjamini-Hochberg, [38]).

**Fig 1:**
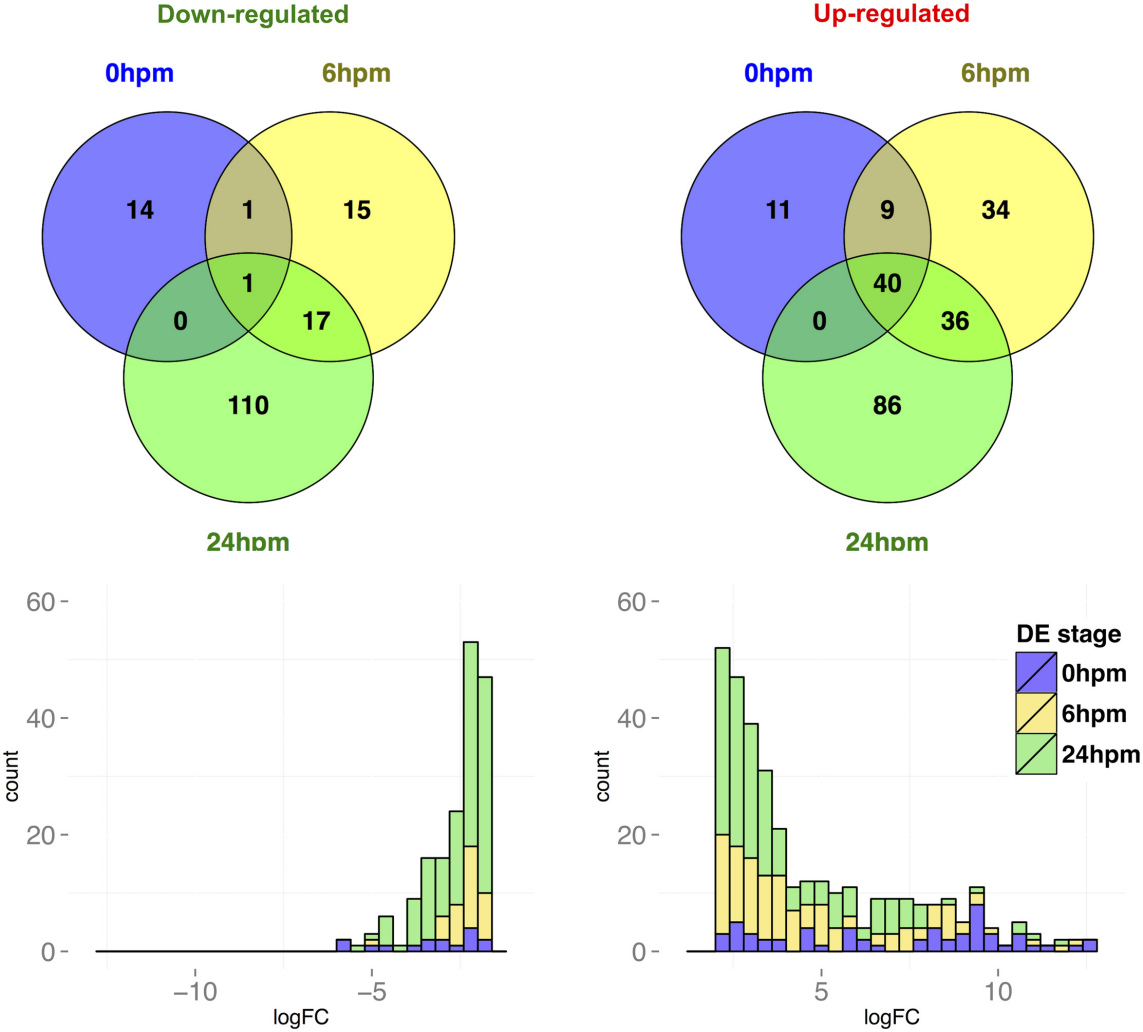
Transcripts that are significantly differentially expressed after mating. (A) Venn diagram showing the number of down-regulated (left) and up-regulated (right) transcripts across the three post-mating time points. The up- and down-regulated gene counts are not mutually exclusive, such that transcripts that are both significantly up- and down-regulated are counted in both sets. (B) Histogram of log2 fold-change for transcripts with 2-fold or higher difference in abundance between virgin and post-mating samples (divided into 0.5 log2 bins). Each bin is partitioned into the time-point in which differential expression is detected (DE stage). The histograms are cumulative, such that transcripts that are differentially expressed in multiple time-points are represented multiple times.

When considering differential expression across all three post-mating time points, we found a total of 364 transcripts to be significantly differentially expressed between virgin and post-mating RT samples (Fig 1A). The median fold-change among transcripts that are significantly up- and down-regulated across the three time-points was approximately 12-fold and 6-fold, respectively. The highest increases in abundance were observed at 0hpm (median fold-change ≈275 fold) whereas the increases in abundance at 6hpm and 24hpm were markedly lower (median fold-change ≈13-fold and 9-fold, respectively). Furthermore, several of the transcripts that increase at 0hpm maintain high abundance at later time-points. On the other hand, the median decrease in abundance across the three post-mating time-points was of lower magnitude than the increase in abundance, ranging from 4.5-fold to ∼8-fold. Overall, the majority of differentially expressed transcripts were found in the 24hpm time-point (Fig 1).

To glean information about alternative splicing, we also examined our data for changes in isoform abundance. However, isoform discrimination relies on the relatively small number of reads that span specific exon-exon boundaries, and therefore the subset of isoforms for which we had sufficient data to draw conclusions was lower than for total gene expression. Nevertheless, several interesting examples were identified where dramatic changes in relative isoform abundances are detected across the mating time course (S4 Fig). Broadly, the isoforms’ behaviors mirror those seen in the overall transcriptome data, insomuch as the abundance of some isoforms increases at specific time points, while others decrease. A further analysis of the splicing changes that accompany changes in post-mating gene expression will require deeper sequencing and/or improved techniques to fully characterize changes in alternative splicing.

### Validation of RNAseq results by qRT-PCR

**Fig 2:**
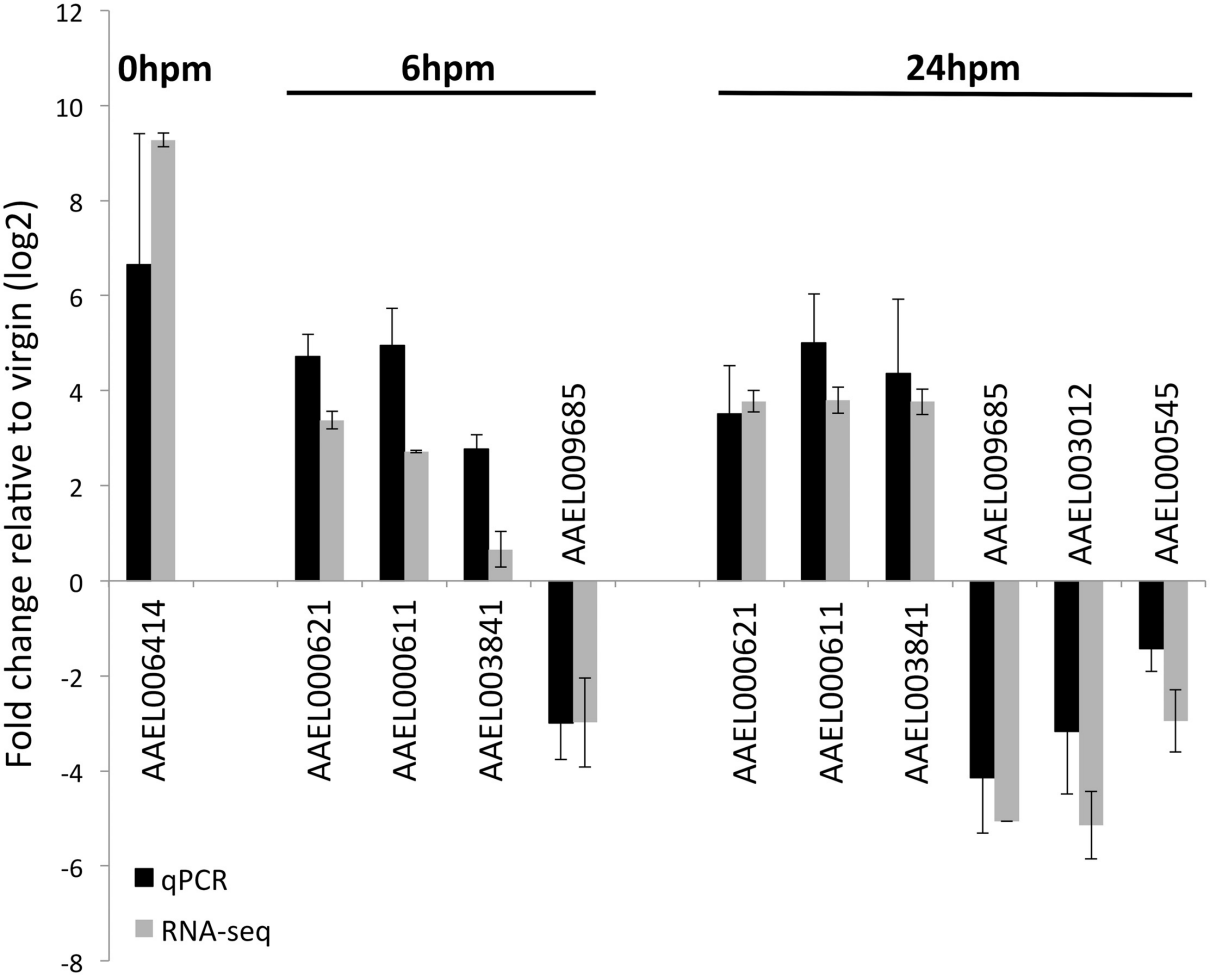
Expression patterns of seven genes using quantitative RT-PCR. Each sample was obtained from the female reproductive tract minus the ovaries at different time points after mating. Each sample represents three different biological replicates, two of them using the expression of the gene *RpS17* for normalization and a third using *actin* expression. Black bars show the results of the quantitative PCR and gray bars show the results of the RNAseq data. Error bars represent standard deviation. A Pearson correlation coefficient shows a positive correlation between qRT-PCR and RNAseq results (*R*^2^ = 0.912, *p*-value = 4.88E-6).

To confirm the validity of differential expression results from the RNAseq analysis, we extracted new samples of RNA and used qRT-PCR to examine expression levels of seven genes selected based on their expression pattern in the RNAseq dataset (Fig 2). The seven genes include a predicted trypsin (AAEL006414), an oxidoreductase (AAEL009685), two cecropins (AAEL000621 and AAEL000611), a zinc metalloprotease (AAEL003012), a defensin (AAEL003841) and a gene with unknown function (AAEL000545). Melt-curve analysis revealed a single product for each tested gene, confirming the efficacy of primer amplification. In general, the fold change estimates from both analyses were in close agreement (Fig 2). Our qRT-PCR validated the reliability of our RNAseq data. For all seven transcripts, the direction of fold-change was the same in the qRT-PCR and the RNAseq analysis. Moreover, the magnitude of fold-change for any given mRNA in both analyses is within 2 standard deviations.

### Many transcripts change in abundance immediately after mating (0hpm)

**Fig 3:**
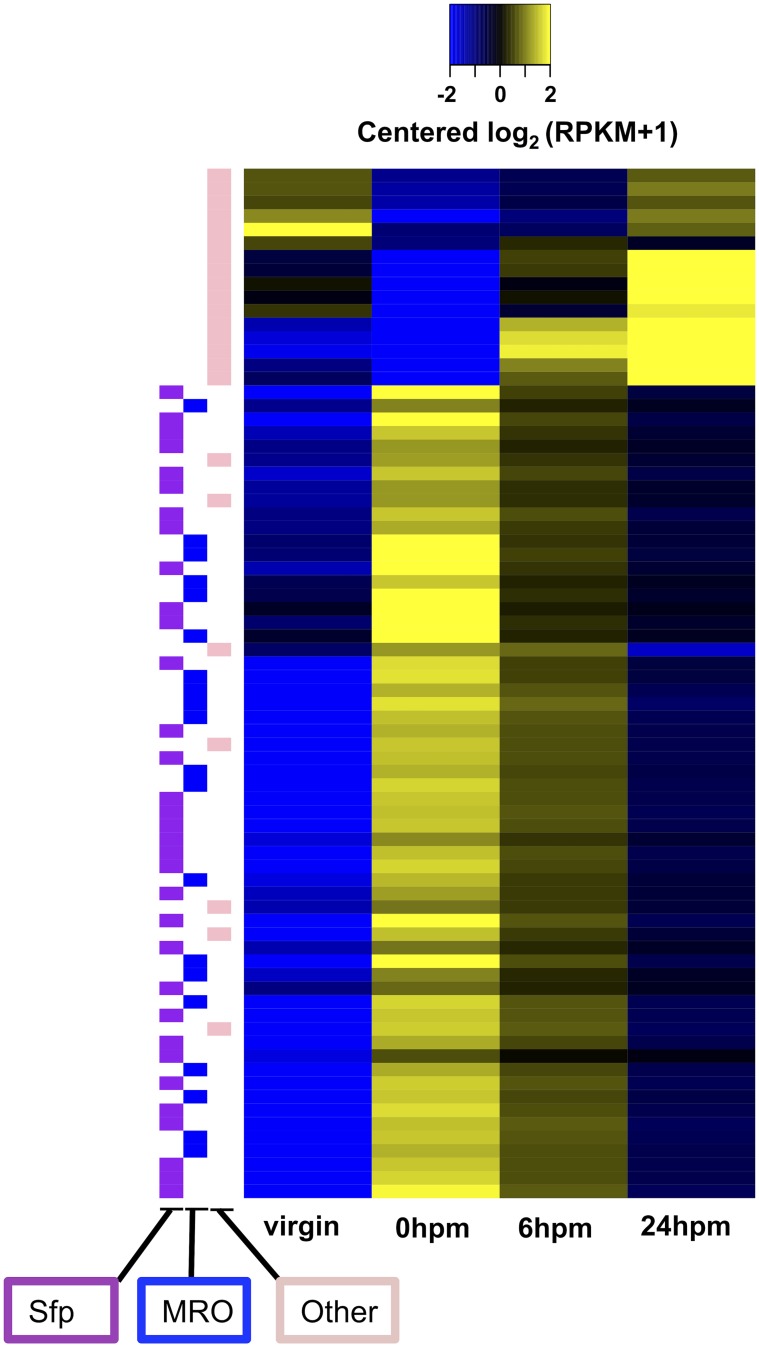
Expression profile of transcripts that are differentially expressed between the virgin and 0hpm sample, including their behaviors at 6 and 24hpm. The color scale represents the median-centered log2 RPKM values. Each row is a transcript and the samples are indicated at the bottom (ordered chronologically from left to right). The top 16 transcripts represent the down-regulated set, while the remaining 60 are those with higher abundances at 0hpm relative to virgin. Transcripts found to be up-regulated in male reproductive organs (MRO)[29], as well as known Sfp genes (Sfp)[39], are indicated on the left by blue (n = 20) and purple markers (n = 33), respectively.

**Fig 4:**
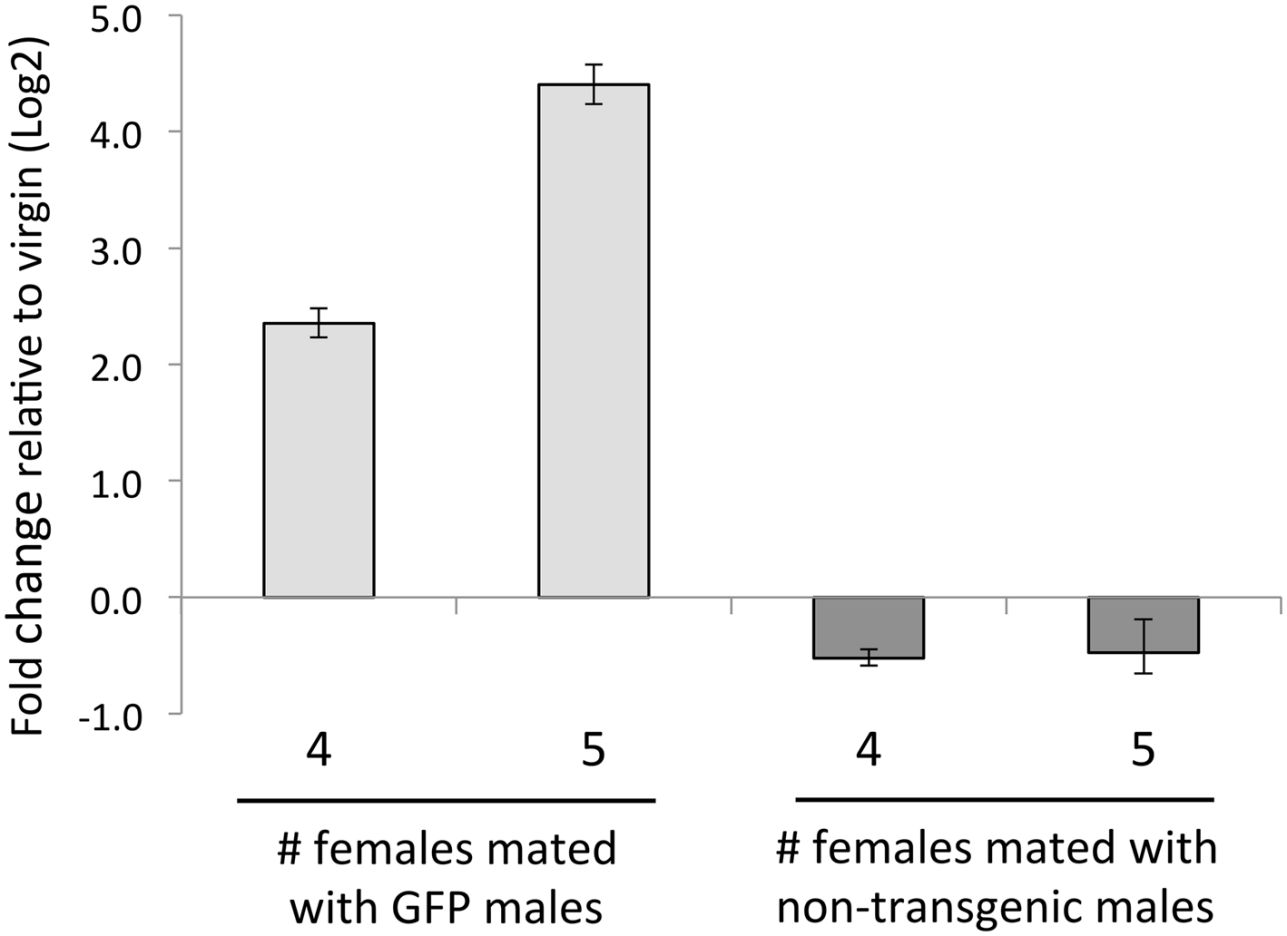
Transfer of eGFP mRNA during mating. GFP mRNA content in wild type females was measure by qRT-PCR. Each sample was obtained from either wild type Thai females (4 or 5 individuals) mated to AAEL010824-GFP transgenic males or wild type Thai females (4 or 5 individuals) mated to non-transgenic males. Relative expression values were calculated by normalizing the expression with *RpS17*. This graph represents three technical replicates, with the error bars representing the standard deviation between those three replicates.

In our comparison of RT transcript levels between virgin females and females dissected immediately after mating (0hpm), we observed 76 transcripts whose abundance was >2-fold different between treatments (Fig 3). Sixteen of these RNAs decreased in abundance (between ∼4-fold and ∼61-fold) in the 0hpm sample relative to the virgin sample, but nearly all of them gradually increase in abundance in later time points; these include RNAs encoding several antimicrobial peptides. On the other hand, sixty transcripts showed increased abundance (between ∼5-fold and ∼1.4x10^4^-fold) at 0hpm, with a median fold change of 275. These include 53 transcripts that are highly expressed in male reproductive tissues [29] (S5 Fig, Supplementary Results). Twenty of these transcripts encode known *Ae. aegypti* Sfps [39], including the previously described Sfp AAEL010824 [35]. The transfer of Sfps seen here is similar to that reported in *D. mojavensis*[34]. However, likely due to strong selection on Sfps in general [40], we found no clear homology between Sfp transcripts transferred in *D. mojavensis* and *Ae. aegypti*. Next, using RT-qPCR, we detected eGFP mRNA in the RT of wildtype females mated to AAEL010824-GFP transgenic males (Fig 4). These data, and the fact that new transcripts are highly unlikely to have been induced in the females in such quantity during the short time-frame of an *Ae. aegypti* mating, suggest that the RNAs detected as “up-regulated” at 0hpm were likely male-derived.

The expression levels of most of these allegedly male-derived transcripts follow a similar trend of exponential decay, implying that these transcripts are transferred to the female in one pulse and are then degraded at a uniform rate. One dramatic case is an unannotated transcript (XLOC019584) that has the highest abundance level at 0hpm (1.76 × 10^4^ RPKM) and appears to be specifically expressed in Thai strain males, but not expressed in the Liverpool strain [29]. Mass spectrometry of male accessory gland extracts from the Thai strain (Villarreal, Avila, Wolfner, and Harrington, *in prep*) identified three peptides produced from isoforms of this mRNA (S9 Fig), consistent with XLOC019584 encoding a seminal protein. It is possible that the male-derived transcripts serve no function in the female, and are simply membrane or vesicle-associated byproducts of apocrine secretion from the male accessory glands.

### Differential expression at 6hpm and 24hpm

**Fig 5:**
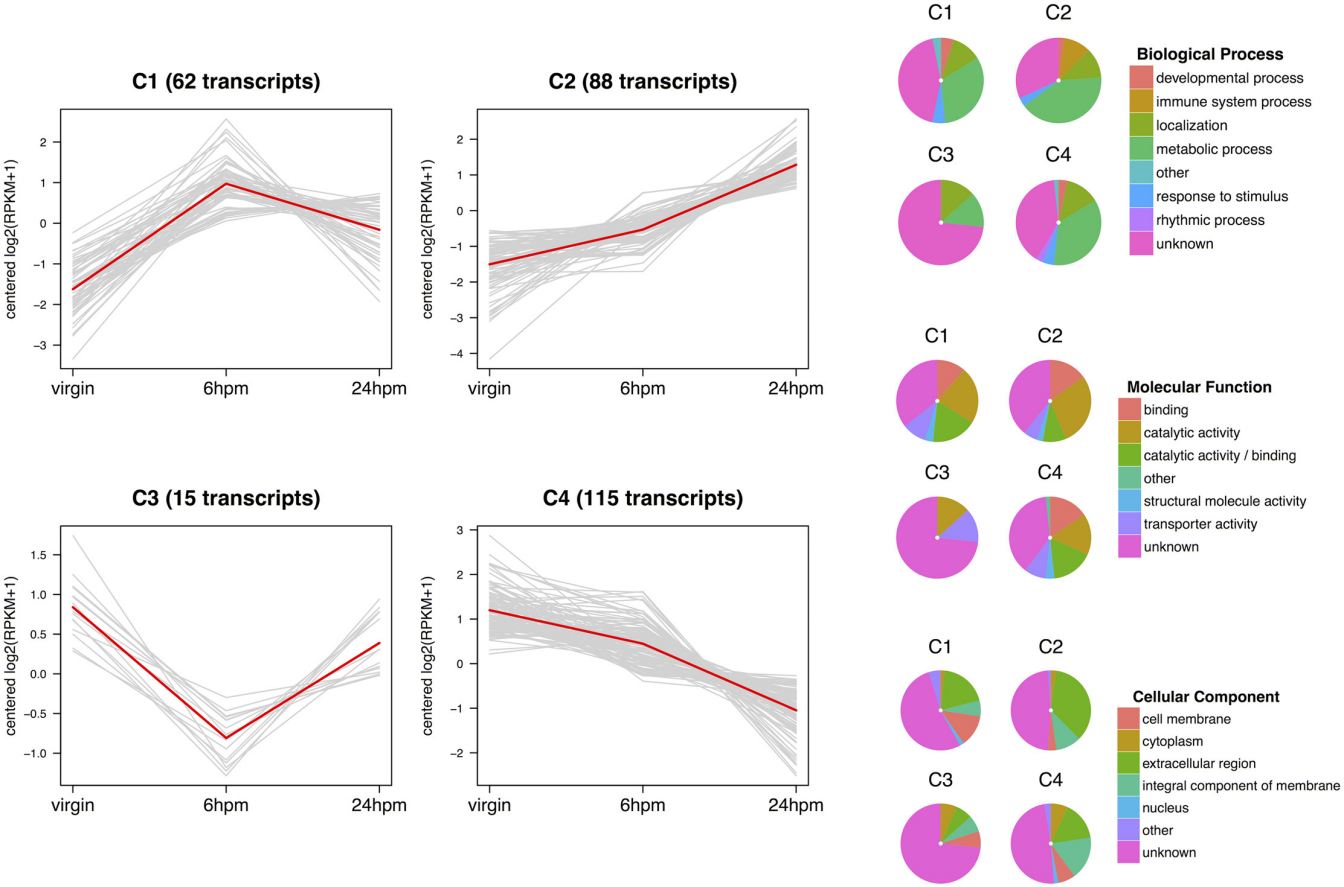
Transcripts that are significantly up- or down-regulated at 6 and 24hpm. (A) Merged clusters from the *K*-means clustering analysis depicting the four mean expression profiles (red line) among transcripts differentially expressed between virgin and later time-points (6hpm and 24hpm). (B) Pie charts of GO terms associated with up-regulated transcripts (C1 and C2) and down-regulated transcripts (C3 and C4). Only ancestral GO terms are shown for the three ontology classes.

After removal of allegedly male-derived transcripts (60 transcripts), we found 280 transcripts that were significantly up- or down-regulated between virgin and the later post-mating (6hpm and 24hpm) samples (S6 Fig). Because of the correction for multiple tests, the number of significantly differentially expressed transcripts in this second analysis is less than the number obtained if the number of male-derived transcripts were simply subtracted from the original number of differentially expressed transcripts (364). To examine the expression profiles and functional classes of transcripts that are differentially expressed at 6hpm and 24hpm, we clustered transcripts into groups by *K*-means clustering. First, we estimated *K* as the square root of half the number of transcripts [41], which yielded 12 clusters. Second, clusters with similar mean expression profile were merged (S7 Fig), such that peak abundance at any given time point is similar. We ultimately obtained a set of four clusters that summarize the observed variation in expression profiles among the differentially expressed transcripts (Fig 5A, S3 Table).

It has recently been shown that some genes in *Ae. aegypti* are under circadian regulatory control [42]. Because these patterns can influence influence interpretation of abundance changes in our study, we examined the circadian expression profile of the 280 transcripts identified here and found that only 10 of these show a circadian pattern of expression (S10 Fig). Therefore, the expression changes we report here are largely due to mating.

### Transcripts up-regulated by mating

To better understand the biological significance of the changes we detected, we carried out GO enrichment analysis of the 150 transcripts that increased in abundance between virgin and mated-female RTs at 6hpm and 24hpm as a whole (Table 1) and as partitioned into the two clusters depicting different expression profiles (C1 and C2, Fig 5A, S2 Table). We also assigned GO terms for each transcript using Ensmbl and VectorBase (Fig 5B, S3 Table). Approximately 37% of these transcripts (57) encode proteins of unknown function (i.e. not classified as belonging to any specific biological process) (Fig 5B, S3 Table). The remaining transcripts largely encode proteins involved in cellular localization (17), proteolysis (26), and immunity (9). The latter were among the most differentially expressed, particularly at 24hpm; their mean increase in expression was ∼10-fold. Further, the largest categories of up-regulated genes with predicted function were those with binding (20) and peptidase activity (39) (S3 Table).

**Table 1:** Gene ontology (GO) enrichment analysis for up- and down-regulated genes.

DE class	Category	GO term	DE trans. (total)	*p*-value	FDR
Up-regulated	Biological Process	proteolysis	26 (840)	7.10E-09	1.90E-05
		innate immune response	12 (212)	2.10E-07	3.10E-04
		immune response	13 (272)	4.00E-07	4.80E-04
		protein processing	11 (200)	1.10E-06	1.30E-03
		protein maturation	11 (206)	1.50E-06	1.60E-03
		defense response to other organism	11 (220)	2.50E-06	2.40E-03
		zymogen activation	8 (110)	4.70E-06	4.20E-03
		maternal specification of dorsal/ventral axis, oocyte, germ-line encoded	8 (115)	7.10E-06	6.00E-03
		defense response	14 (410)	8.00E-06	6.00E-03
		defense response to bacterium	9 (173)	1.60E-05	1.00E-02
		serine family amino acid metabolic process	5 (39)	2.10E-05	1.30E-02
		oocyte dorsal/ventral axis specification	8 (139)	2.40E-05	1.40E-02
		Toll signaling pathway	8 (139)	2.60E-05	1.50E-02
		response to bacterium	9 (186)	2.80E-05	1.50E-02
		response to other organism	11 (294)	3.20E-05	1.60E-02
		sterol transport	5 (44)	3.40E-05	1.70E-02
		immune system process	13 (418)	3.70E-05	1.80E-02
		oocyte axis specification	8 (152)	4.20E-05	1.90E-02
		alpha-amino acid metabolic process	9 (196)	4.70E-05	2.10E-02
		response to external stimulus	17 (701)	5.30E-05	2.30E-02
		response to external biotic stimulus	11 (329)	8.70E-05	3.50E-02
		response to biotic stimulus	11 (332)	9.50E-05	3.70E-02
		dorsal/ventral axis specification	8 (171)	1.00E-04	3.70E-02
		cholesterol efflux	4 (30)	1.10E-04	3.90E-02
	Molecular Function	peptidase activity, acting on L-amino acid peptides	30 (963)	1.50E-10	1.00E-06
		peptidase activity	30 (991)	3.00E-10	1.40E-06
		endopeptidase activity	25 (740)	1.30E-09	4.40E-06
		serine-type endopeptidase activity	18 (419)	1.80E-08	4.00E-05
		serine-type peptidase activity	18 (457)	6.70E-08	1.30E-04
		serine hydrolase activity	18 (465)	8.70E-08	1.50E-04
		hydrolase activity	44 (2541)	3.40E-07	4.60E-04
		sterol transporter activity	4 (16)	8.50E-06	6.00E-03
		cholesterol transporter activity	4 (16)	8.50E-06	6.00E-03
		lipid transporter activity	6 (63)	9.80E-06	6.60E-03
		glycine dehydrogenase (decarboxylating) activity	2 (2)	6.50E-05	2.60E-02
		oxidoreductase activity, acting on the CH-NH2 group of donors, disulfide as acceptor	2 (2)	6.50E-05	2.60E-02
		substrate-specific transporter activity	17 (725)	9.80E-05	3.70E-02
		metalloendopeptidase activity	6 (94)	1.40E-04	4.70E-02
	Cellular Component	extracellular region	41 (1220)	2.20E-15	2.90E-11
Down-regulated	Molecular Function	carboxypeptidase activity	6 (49)	2.80E-07	2.00E-03
		metallopeptidase activity	10 (214)	4.20E-07	2.00E-03
		exopeptidase activity	9 (164)	4.50E-07	2.00E-03
		peptidyl-dipeptidase activity	3 (8)	1.40E-05	4.50E-02

Two clusters derived from the *K*-means approach comprise the up-regulated set (Fig 5A). They are characterized by the time-point in which the fold-change was highest: Cluster 1 contains transcripts that are most abundant at 6hpm, while cluster 2 contains transcripts whose abundance was highest at 24hpm.

#### Transcripts with highest abundance at 6hpm (Cluster 1)

Among the 150 up-regulated genes, 62 had their highest abundance levels in the female RT at 6hpm relative to virgins (Fig 5A). Among the molecular functions of this gene set, RNAs encoding predicted catalytic proteins (24 transcripts) and binding enzymes (17 transcripts) formed the largest group (Fig 5B, S3 Table). These catalytic enzymes are largely composed of proteases (10 transcripts), but also include ATPases (6 transcripts), oxidoreductases (3 transcripts), and various hydrolases. The significantly enriched GO terms in cluster 1 include sterol transport and cholesterol transporter activity (S2 Table).

Several peptidases are found in this cluster, particularly those with serine-type endopeptidase activity. This result parallels findings in *D. melanogaster*[21], where several protease-encoding RNAs are up-regulated in the female RT after mating. Two of the serine-type endopeptidases in this cluster are predicted oviductins (AAEL014567 and AAEL014570). In *Xenopus laevis*[43] and *Bufo japonica*[44], oviductins modify egg envelopes during passage through the oviduct, preparing them for fertilization. Oviductins also play a role in facilitating sperm binding and sperm-egg interactions. However, fertilization in mosquitoes differs from that in vertebrates, so the potential role of these oviductins in *Aedes* reproduction is not clear. Two other predicted serine-type endopeptidases in cluster 1 belong to the Clip-domain serine protease family (AAEL002997 and AAEL007993). Members of this family play roles in early embryonic polarity of *Drosophila* through activation of the Toll pathway [45], as well as in the activation of immune processes in insects [46].

Eight additional predicted proteases (AAEL009843, AAEL008567, AAEL005666, AAEL001703, AAEL009853, AAEL010139, AAEL008781 and AAEL002661) are found in cluster 1. Most of these proteases are ubiquitously expressed in many organisms and may play roles in immunity, fertilization, development, and apoptosis [47]. Two of them (AAEL005666 and AAEL002661) encode matrix metalloproteinase 14 and 19. Metalloproteases, which hydrolyze components of the extracellular matrix and allow matrix and tissue remodeling, play roles in several reproductive processes in mammals [48]. A recent study in *Drosophila* shows that matrix metalloproteinase 2 (*mm2*) is required for ovulation by degrading the follicle cells surrounding the egg as the latter enters the oviduct [49].

Some cluster 1 transcripts derive from genes that may be involved in priming the female for oogenesis and egg development or other post-mating functions. One such gene encodes the folate transporter 1 (XLOC001733). Folate metabolism is important for the production of functional oocytes in *Drosophila* and *Caenorhabditis elegans*[50, 51], and it is tempting to speculate that XLOC001733 might be important in folate uptake or homeostasis for oogenesis in *Ae. aegypti*. Another gene in this cluster encodes lipase 3 (AAEL015326). Lipases are important for the utilization of lipids as energy source in many species. In the sand fly, *Phlebotomus papatasi*, a lipase-like protein constitutes a major component of the female accessory gland secretion [52], while in the moth, *Manduca sexta*, lipoprotein lipase activity was observed in the ovarian follicles [53]. Additional cluster 1 transcripts in the oogenesis/development group encode proteins predicted to suppress cytokine signaling (AAEL000393) and the Janus kinase hopscotch (AAEL012553), both of which are involved in the JAK/STAT signaling pathway. JAK/STAT signaling has been associated with stem cell activation in *Ae. aegypti*[54] and is required for oogenesis in *Drosophila*[55]. Additionally, within this cluster we found a transferrin (AAEL012949). In *Ae. aegypti*, transferrins have been linked to egg development [56]. Expression of transferrin in other insects also suggests reproductive functions. In the tsetse fly, transferrin was detected in testes and females’ spermathecae [57]. Additionally, in *Sarcophaga peregrine*, transferrin is involved in transporting iron into eggs during oogenesis [58].

Several genes with potential immune function were found to be induced at 6hpm. One such gene is the previously mentioned transferrin. Transferrins bind iron ions, which are often a limiting nutrient for bacteria. Indeed, a transferrin has been shown to be up-regulated in response to bacterial and filarial worm infection, suggesting that induction of transferrin RNAs might also contribute to immune function in *Ae. aegypti*[56]. These proteins may serve to protect the female from bacterial infection in the face of an imminent blood meal, which provides iron and other nutrients that could otherwise spur on bacterial infection. Those genes involved in the JAK/STAT pathway may also induce post-mating immune responses [54]. Finally, two predicted serine-type endopeptidases in Cluster 1 belong to the Clip-domain serine protease family (AAEL002997 and AAEL007993) and may activate immune processes in insects [46].

In comparison with RNA levels in virgins, genes with immune function were modulated at all three post-mating time points. Several anti-microbial peptide (AMP) transcripts are induced at later time points and show greatest expression at 24hpm, where genes with immune function are significantly up-regulated (S2 Table). This surplus of AMP transcripts may be priming the immune system to ward off venereally transmitted pathogens. An increase in AMP production in response to mating has previously been shown in *D. melanogaster*, where mating induces an increase in the expression of genes encoding AMPs [13, 14, 24]. However, in other organisms, such as *An. gambiae*[20] and the medfly *Ceratitis capitata*[19], a significant post-mating increase in levels of RNAs encoding AMPs was not reported (but see below).

#### Transcripts with highest abundance at 24hpm (Cluster 2)

Similar to results reported for *An. gambiae*[20, 23], we found the highest number of genes induced in the female RT at 24hpm, indicating that mating triggers broad transcriptional cascades at least up until this time point; 88 of these reach their highest abundance at 24hpm. As in cluster 1, many of the transcripts up-regulated at this time encode proteins with predicted proteolysis function (19%), as well as proteins with roles in the innate immune response (11%), and these are highly significantly enriched (S2 Table). Further, 6 genes encoding metalloproteases were among the highest expressed at this time (AAEL011292, AAEL006768, AAEL006777, AAEL006766, AAEL006761 and AAEL012873), including the angiotensin-converting enzyme (ACE; AAEL012873), whose ortholog’s expression is induced by a blood-meal in female *Anopheles stephensi*[59]. In the developing ovaries, ACE accumulates until eventually moving into mature eggs, where it may facilitate embryonic peptide metabolism [59]. In the moth *Lacanobia oleracea*, ACE activity was detected in male accessory glands and the female bursa and spermathecae [60].

Cluster 2 also contains many transcripts that are potentially important in further priming the female reproductive response, as well as transcripts of genes with predicted immune function. Two transcripts coding for gamma glutamyl transpeptidases (AAEL010936 and AAEL015561) and 2 vitellogenin transcripts (AAEL010434 and AAEL006138) were up-regulated at 24hpm. A gamma glutamyl transpeptidase has been found in *Ae. aegypti* eggshell preparations [61] and in *Drosophila* male accessory glands [62], whereas vitellogenin serves as a primary egg provisioning protein [63]. Vitellogenins may also play a role in immunity. Recent work in honeybees has demonstrated an essential role of vitellogenin in conveying segments of bacteria into eggs, potentially serving as a mechanism for transgenerational immunity [64]. It is possible, however, that the increase in vitellogenin abundance in the RT reflects the dramatic increase of these transcripts in the fat body after mating and/or intake of a blood meal [28] (S8 Fig).

Finally, three transferrin genes (AAEL015458, AAEL015639 and XLOC005057) showed increased transcript levels at 24hpm. As discussed previously, transferrins play a role in iron metabolism, immunity and development [65]. Transferrin 2 (AAEL015639) is highly expressed in adult female ovaries 72h after a blood meal, suggesting a role in egg development, while transferrin 1 (AAEL015458) is up-regulated upon a bacterial infection, suggesting a role in immune response [56]. A gene that is orthologous to these transferrins in *An. gambiae* (AGAP000376) is similarly upregulated at 24hpm [23] (S4 Table, S11 Fig). Several AMPs are also higher in abundance at 24hpm (Table 1, S2 and S3 Tables), which include defensins (AAEL003841, AAEL003857, AAEL003832 and AAEL003821) and cecropins (AAEL000611, AAEL000621, AAEL018349 and AAEL015515). We also found that the orthologues of these genes in *An. gambiae* are upregulated in the RT at 24hpm, suggesting a possibly conserved immune response across mosquitoes [23] (S4 Table, S11 Fig).

Other transcripts up-regulated at 24hpm include Cathepsin I (AAEL002833) [65], prophenoloxidase (AAEL013492) [66], and C-type lectins (AAEL018265 and AAEL011610). These genes play a role in the innate immune response in mosquitoes and other insects (reviewed in [67]). The Cathepsin I orthologue in *An. gambiae* (AGAP011828) also increases in expression after-mating [23] (S4 Table, S11 Fig). Lectins serve other documented functions in dipterans, including sperm maintenance and release in *D. melanogaster*[68] and facilitation of some arboviral infections in *Ae. aegypti*[69].

### Transcripts down-regulated by mating

130 transcripts were down-regulated relative to virgin and mated females at 6hpm and 24hpm. Of these, 15 were down-regulated at 6hpm and 115 were down-regulated at 24hpm. The latter set contained 32 previously unannotated transcripts. GO analysis of the down-regulated transcripts shows that 44% (55 transcripts) are not classified as belonging to a specific biological process. The remaining transcripts encode proteins involved in binding (16 transcripts) and catalytic activity (40 transcripts) (Fig 5B, S3 Table). Two clusters derived from the *K*-means approach represent transcripts that have lowest abundance at 6 and 24hpm (Fig 5A).

#### Transcripts with the lowest abundance at 6hpm (Cluster 3)

Fifteen transcripts show lowest abundance levels at 6hpm. Two of these encode proteins involved in transporter activity: a norepinephrine transporter (AAEL005581) and a cyclic nucleotide-gated cation channel (AAEL011638). The norepinephrine transporter is orthologous to the *D. melanogaster* *serT* gene, which regulates serotonin action by re-uptake of the extracellular neurotransmitter [70]. In *Locusta migratoria*, serotonin regulates oviduct muscle contraction [71]. In *D. melanogaster*, serotonin is re-localized to the ovary and seminal receptacle immediately after mating. This may indicate the rapid uptake of the neurotransmitter into nerve termini by transporters [72]. Other studies have shown that the female nervous system plays an important role in post-mating responses in *D. melanogaster* (e.g. [73–78]).

#### Transcripts with lowest abundance at 24hpm (Cluster 4)

At 24hpm, 115 transcripts are down-regulated relative to virgin females. This cluster of transcripts consists of transcripts that are gradually down-regulated from 0hpm to 24hpm. This cluster is also characterized by a wide variety of gene functions and biological processes. Several down-regulated RNAs at 24hpm encode proteins involved in proteolysis (11%) and oxidation-reduction processes (8%) (S3 Table). In contrast to up-regulated transcripts, the significantly enriched GO terms were carboxypeptidase, metallopeptidase, exopeptidase and peptidyl-dipeptidase activity (Table 1, S2 Table). Remarkably, the latter GO terms are also characteristic of the set of down-regulated transcripts in *An. gambiae* at 24hpm [23], suggesting a conserved process by which resources are reallocated from general metabolism to reproduction.

Among the down-regulated RNAs predicted to encode proteins with proteolytic activity, we found the exopeptidase class, such as carboxypeptidase (AAEL010782, AAEL010776 and AAEL012351), angiotensin-converting enzyme (AAEL017460, AAEL009310 and AAEL009316) and metalloproteinase (AAEL008163, AAEL002655 and AAEL003012) to be enriched. Angiotensin-converting enzyme (ACE) is widely distributed in many insects and in a variety of tissues, suggesting diverse biological roles [79]. Besides the previously discussed role for ACE in egg laying (in *A. stephensi*), these proteins are important in the reproduction of the Egyptian cotton leafworm; ACE inhibition results in decreased oviposition [80, 81].

A second set of down-regulated transcripts play a role in oxidation-reduction processes. These include RNAs from chorion peroxidase genes (AAEL000496 and AAEL000507), a glucose dehydrogenase (AAEL011806) and an aldehyde oxidase (AAEL010367). *Ae. aegypti* chorion peroxidases play a role in egg chorion hardening [82]. *D. melanogaster* glucose dehydrogenase is secreted into the lumen of the spermathecal ducts and is necessary for sperm storage [83]. Finally, aldehyde oxidase has been linked to immune response in *Ae. aegypti*[84].

The fact that transcripts with oxidation-reduction function were significantly down-regulated was unexpected. Given that an impending blood meal will introduce reactive oxygen species, which could damage sperm [27] or the female reproductive tract, we expected to find this class to be up-regulated after mating, as has been shown in *An. gambiae* in response to a blood meal. Perhaps protective enzymes are not induced until a blood meal is ingested, or are expressed in other tissues. Undoubtedly, however, some genes are induced that assist in the storage and maintenance of sperm in the spermathecae. In *D. melanogaster*, a lectin has been shown to be necessary for proper sperm storage [85], and our data reveal RNAs encoding two lectins (AAEL018265 and AAEL011610) that are up-regulated in the female reproductive tract at 24hpm and may serve a similar function.

Other down-regulated genes at 24hpm encode proteins involved in lipid metabolism and transport, such as elongase (AAEL011957) and the CRAL/TRIO domain-containing protein (AAEL005779). In *Ae. aegypti*, a large amount of lipids are found in oocytes and they accumulate after a blood meal, suggesting that lipid metabolism genes begin to be turned off due to the lack of blood meal [86].

Transcripts of three genes thought to regulate juvenile hormone are also down-regulated at 24hpm: a GPCR Galanin/Allatostatin family protein (AAEL012920), Venom carboxylesterase-6 (juvenile hormone esterase 6, AAEL012886) and Juvenile hormone esterase (AAEL000545). This fits with what is known about the tight regulation of JH levels during the gonotrophic cycle; in female mosquitoes, high JH levels are required during the stages leading up to blood digestion and oogenesis. JH levels then drop to low levels during digestion of the blood and subsequent vitellogenesis [87].

### Conclusions

*Aedes aegypti* females typically mate near the host, after which they take a blood meal and ultimately develop eggs. Therefore, we postulate that mating primes a female for these downstream processes, triggering expression changes that physiologically prepare her for blood meal digestion and oogenesis. In contrast with model organisms, such as *D. melanogaster*, relatively few genes in *Aedes aegypti* have had their functions elucidated experimentally. Therefore, we have speculated here about potential roles of genes in the post-mating response based on their putative functions, with the caveat that these suppositions must be tested.

Mating is directly linked to a female’s vectorial capacity via altered blood-feeding behavior and increased reproductive potential. Therefore, a thorough knowledge of mating-induced transcriptional changes may aid in developing tools that aim to prevent disease transmission by manipulating the post-mating response. Our dataset provides a framework by which this crucial life history milestone in *Ae. aegypti* can be examined. This study deepens our understanding of broad-scale gene expression changes after mating, and will serve as a launching point for future gene-specific investigations.

## Supporting Information

S1 Methods Read processing and quality control. Description of sequencing procedure and quality control of Illumina reads before differential expression analysis.
(PDF)

S1 Table Primers. Primer sequences used in real-time PCR to validate the expression profiles of differentially expressed genes identified through RNAseq.
(XLSX)

S2 Table GO analysis. Gene Ontology enrichment analysis for merged *K*-means clusters. No statistically significant GO terms were uncovered for Cluster 3.
(XLSX)

S3 Table DE genes. List of differentially expressed genes, abundance levels (RPKM), and annotation information.
(XLSX)

S4 Table Orthologous post-mating response genes in *Ae. aegypti* and *An. gambiae*. Orthologous genes that were identified in this study and in *An. gambiae*[23], their fold-change and annotation.
(XLSX)

S1 Fig Percentage of rRNA-derived reads in each of the libraries used in this study. rRNA transcripts were identified using the VectorBase annotations (www.vectorbase.com) and checked against *in-silico* rRNA prediction software (rnammer, v. 1.2).
(TIFF)

S2 Fig Replicate comparisons of first sequencing runs. Barplot of the percentage of transcripts that show ≥2-fold transcript abundance between replicates of the same sample in the first sequencing run. The x-axis is the log2 read count.
(TIFF)

S3 Fig Replicate comparisons of resequenced samples. (A) Barplot of the percentage of transcripts that show ≥2-fold transcript abundance between replicates of the same sample. Each replicate here is comprised of pooled reads from both sequencing runs. Only libraries that were resequenced used. The x-axis is the log2 read count. (B) MA Plot of replicate comparisons for each of the resequenced samples.
(TIFF)

S4 Fig Isoform level differences in transcript abundance. Several transcripts with different isoform expression profiles (right) compared to whole transcript expression profiles (left).
(TIFF)

S5 Fig Transcripts up-regulated at 0hpm. (A) Abundance levels of transcripts that increase in abundance at 0hpm compared to the virgin sample. XLOC019584, a suspected male-derived transcript with the highest abundance value in the dataset, is indicated. (B) Abundance profile of transcripts that were found to have higher abundance at 0hpm compared to virgin using two recently published datasets: transcriptome levels of male and female reproductive tissues and carcasses (left, [29]) and transcriptome levels of various external tissues (right, [88]). Both datasets use the Liverpool strain of *Ae. aegypti*. (Car. = carcass; ReO = reproductive organs; AT = abdomincal tip; Ov = ovaries; pre/post = pre- post-blood meal).
(TIFF)

S6 Fig Differentially expressed transcripts at 6hpm and 24hpm. Heatmap of 280 transcripts that are significantly up- or down-regulated at 6 and 24hpm compared to the virgin sample shown according to corresponding merged clusters (see Fig 5).
(TIFF)

S7 Fig *K*-means clusters. Initial *K*-means clusters for virgin versus 6hpm and 24hpm comparisons. Clusters were merged based on expression profile and time-point with maximum/minimum median abundance.
(TIFF)

S8 Fig Regulation pattern of RT transcripts in the fat body of *Ae. aegypti*. A comparison of expression patterns between our DE transcripts and those of Roy *et al*.[28] shows low level concordance among up- (orange bar on cladogram) and down-regulated (tan bar) transcripts in response to mating/blood feeding. Colored bars depicting merged *K*-means clusters from Fig 5A are indicated on the left of the heatmap. Two transcripts with the highest increase in abundance in response to mating/blood meal in the Roy *et al*. dataset are two vitellogenins, and those two are up-regulated in response to mating in our dataset, albeit at much lower levels.
(TIFF)

S9 Fig Mass spectrometry peptides. Amino acid sequences generated from XLOC019584 transcripts that exhibit sequence coverage in the mass spectrometry analysis of *Ae. aegypti* male accessory gland extract.
(TIFF)

S10 Fig Circadian expression pattern of post-mating response genes. Ten of the 280 DE transcripts show cyclical expression pattern that corresponds to light/day changes. *K*-means cluster affiliation of each transcript is shown on the left. Light and Dark are indicated on top of the heatmap by white and black rectangles, respectively.
(TIFF)

S11 Fig Comparison of orthologous post-mating response genes in *Ae. aegypti* and *An. gambiae*. Fold-change estimates of DE transcripts in this study and their ortholgoues in *An. gambiae*[23] are shown and their ancestral Biological Process GO terms are indicated. A linear regression with 95% confidence interval is also shown. The data for this plot can be found in S4 Table.
(TIFF)
