# Supplementary material for: Mating-Induced Transcriptome Changes in the Reproductive Tract of Female Aedes aegypti
Source: PLoS Negl Trop Dis. 2016 Feb 22;10(2):e0004451. doi: 10.1371/journal.pntd.0004451 (PMC4764262; doi:10.1371/journal.pntd.0004451)
Supplement: S1 Methods — Description of sequencing procedure and quality control of Illumina reads before differential expression analysis. (PDF) [file pntd.0004451.s001.pdf]

Supplementary Methods:

RNA sequence reads were obtained in two rounds. In the first round, three replicates from each sample (virgin, 0hpm, 6hpm, 24hpm) were sequenced at moderate depth (≈10 million reads/replicate/sample) on the Illumina platform. The percentage of RNA reads derived from ribosomal RNA (rRNA) transcripts—which were largely filtered during library preparation—was estimated for each sample. Finally, The fold-change between replicates of the same sample was calculated to establish a minimum read count cut-off above which differential expression (DE) between samples is reliable.

First, three of the samples contained higher amounts of rRNA-derived reads than the remaining samples (virgin-1 and virgin-2 ≈11%; 24hpm-3 ≈3%; S1 Fig). The remaining samples contained <0.5%. Furthermore, the number of reads mapping to many transcripts varied greatly between replicates of the same sample, primarily due to low read counts at those transcripts. To identify a read-count threshold for DE analysis, the percentage of transcripts that show higher than 2-fold difference between replicates of the same sample was calculated (S2 Fig). This analysis revealed that, above 180 reads/transcript, less than 5% of transcripts show 2-fold change differences between replicates of the same sample. Thus, a read count of 180 reads/transcript was established as the cut-off for DE analysis.

In the second round of sequencing, only the virgin sample that did not contain higher amounts of rRNA reads (virgin-3) and two of each of the post-mating samples (0hpm-2, 0hpm-3, 6hpm-2, 6hpm-3, 24hpm-1, 24hpm-2) were resequenced at higher depths (~20-40 million reads/replicate/sample). Reads from both rounds of sequencing were combined for each resequenced sample and checked for variability among the two replicates from each sample (S3 Fig).
