# Supplementary figures and images for: Mating-Induced Transcriptome Changes in the Reproductive Tract of Female Aedes aegypti
Source: PLoS Negl Trop Dis. 2016 Feb 22;10(2):e0004451. doi: 10.1371/journal.pntd.0004451 (PMC4764262; doi:10.1371/journal.pntd.0004451)

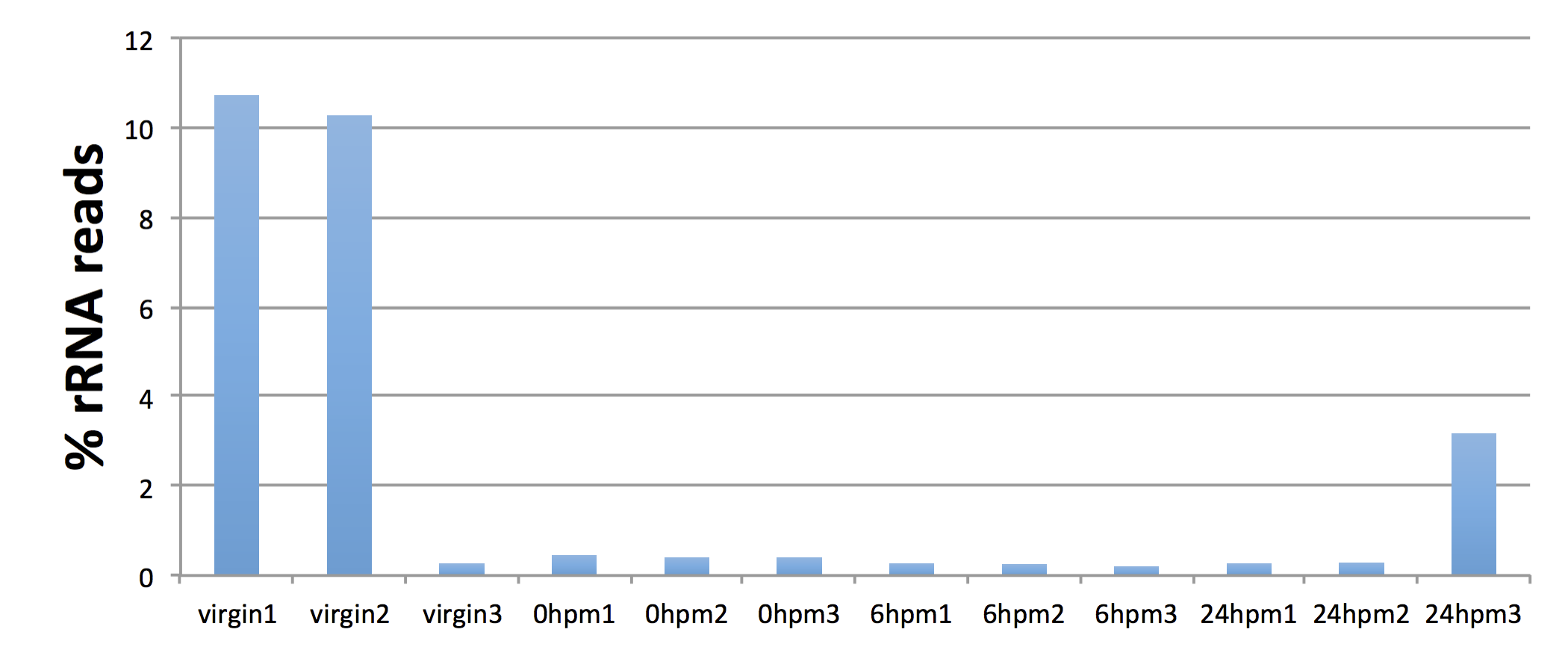

Supplement: S1 Fig — rRNA transcripts were identified using the VectorBase annotations (www.vectorbase.com) and checked against in-silico rRNA prediction software (rnammer, v. 1.2). (TIFF) [file pntd.0004451.s006.tiff]

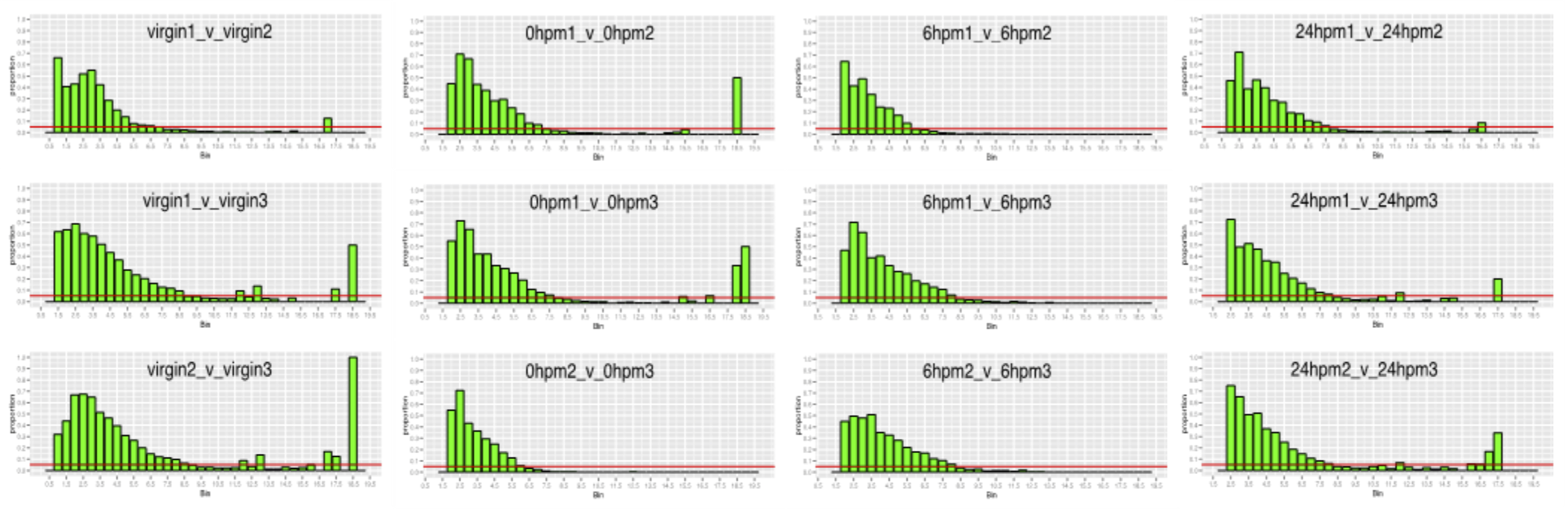

Supplement: S2 Fig — Barplot of the percentage of transcripts that show ≥2-fold transcript abundance between replicates of the same sample in the first sequencing run. The x-axis is the log2 read count. (TIFF) [file pntd.0004451.s007.tiff]

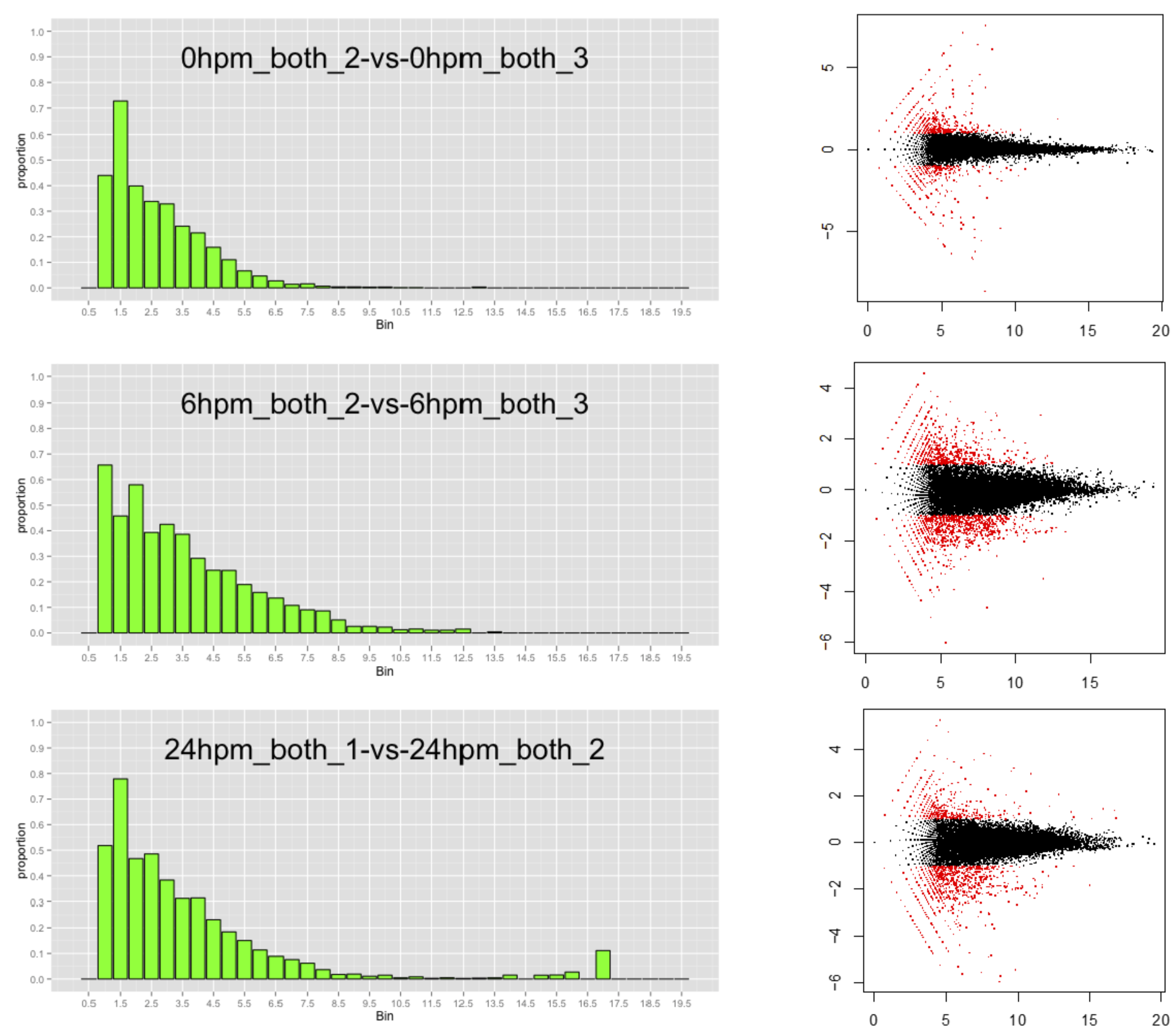

Supplement: S3 Fig — (A) Barplot of the percentage of transcripts that show ≥2-fold transcript abundance between replicates of the same sample. Each replicate here is comprised of pooled reads from both sequencing runs. Only libraries that were resequenced used. The x-axis is the log2 read count. (B) MA Plot of replicate comparisons for each of the resequenced samples. (TIFF) [file pntd.0004451.s008.tiff]

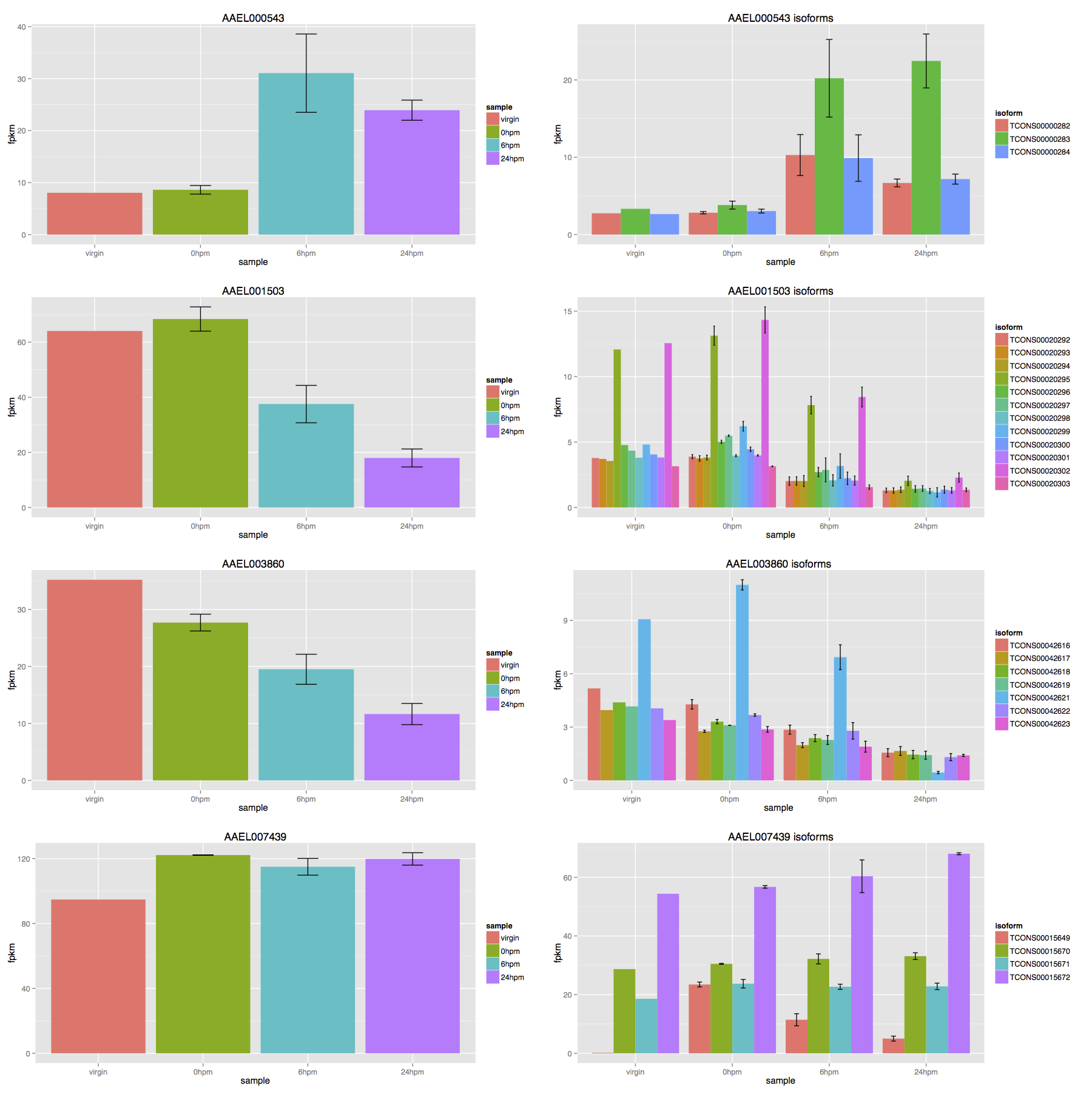

Supplement: S4 Fig — Several transcripts with different isoform expression profiles (right) compared to whole transcript expression profiles (left). (TIFF) [file pntd.0004451.s009.tiff]

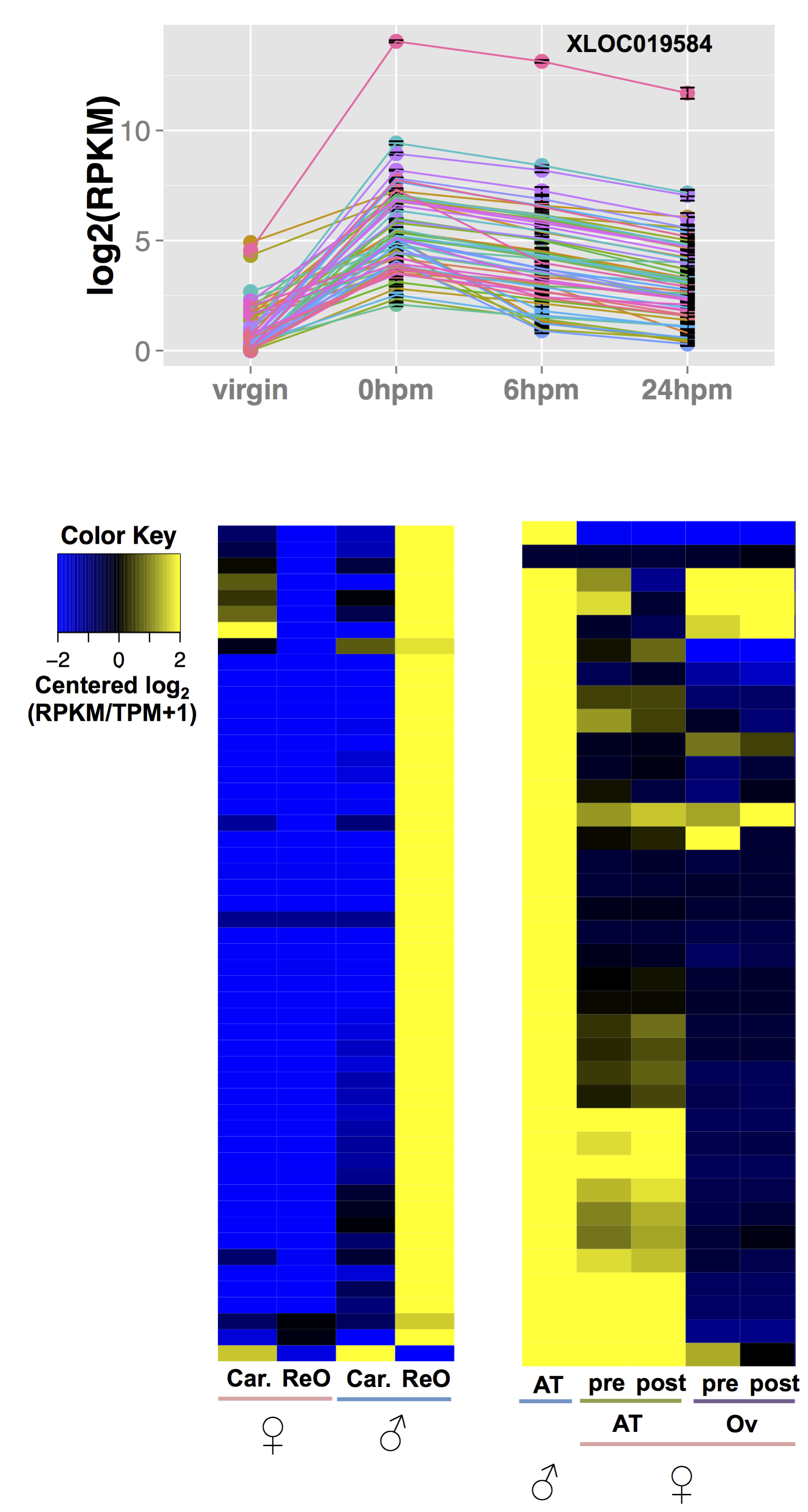

Supplement: S5 Fig — (A) Abundance levels of transcripts that increase in abundance at 0hpm compared to the virgin sample. XLOC019584, a suspected male-derived transcript with the highest abundance value in the dataset, is indicated. (B) Abundance profile of transcripts that were found to have higher abundance at 0hpm compared to virgin using two recently published datasets: transcriptome levels of male and female reproductive tissues and carcasses (left, [29]) and transcriptome levels of various external tissues (right, [88]). Both datasets use the Liverpool strain of Ae. aegypti. (Car. = carcass; ReO = reproductive organs; AT = abdomincal tip; Ov = ovaries; pre/post = pre- post-blood meal). (TIFF) [file pntd.0004451.s010.tiff]

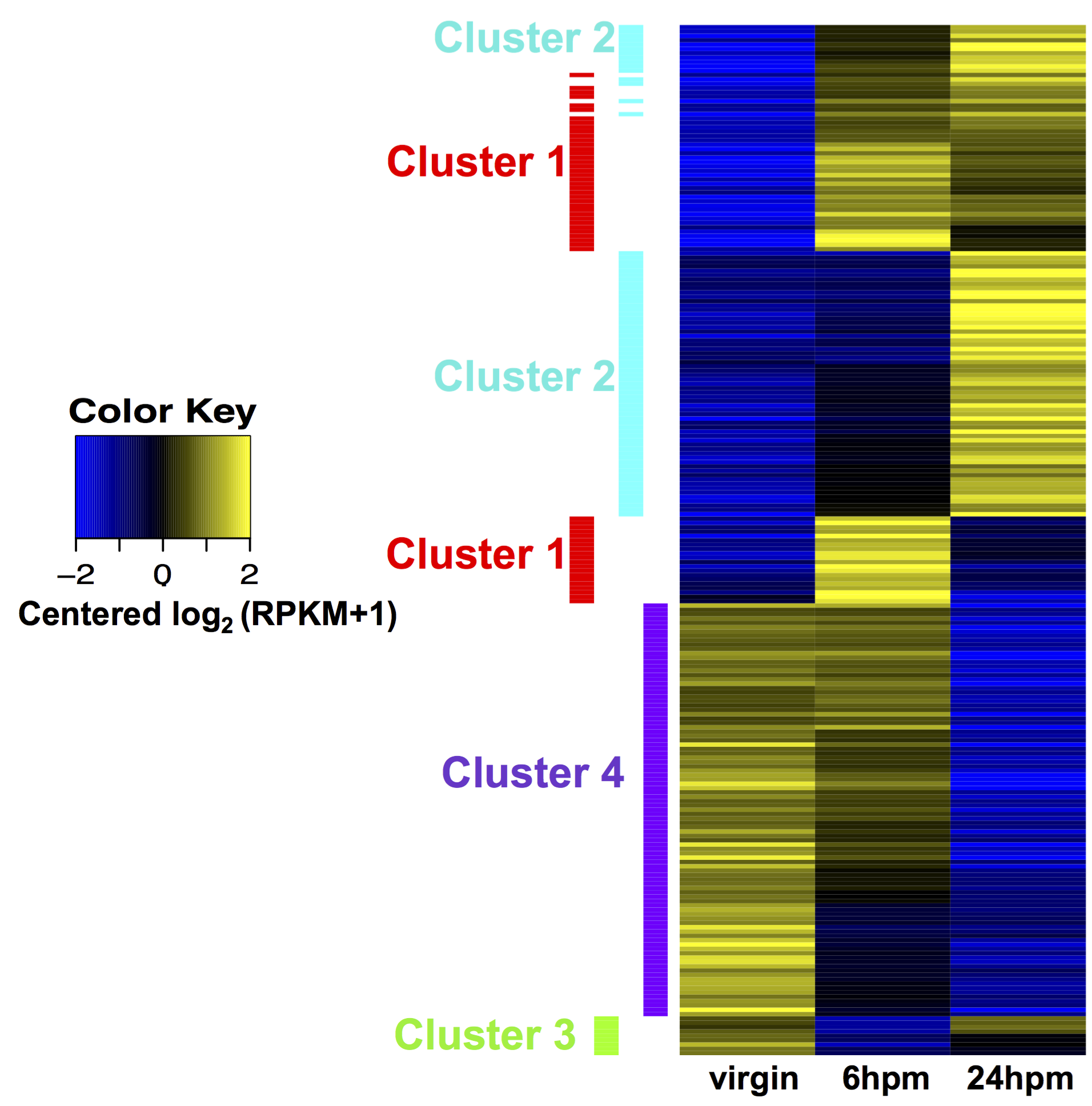

Supplement: S6 Fig — Heatmap of 280 transcripts that are significantly up- or down-regulated at 6 and 24hpm compared to the virgin sample shown according to corresponding merged clusters (see Fig 5). (TIFF) [file pntd.0004451.s011.tiff]

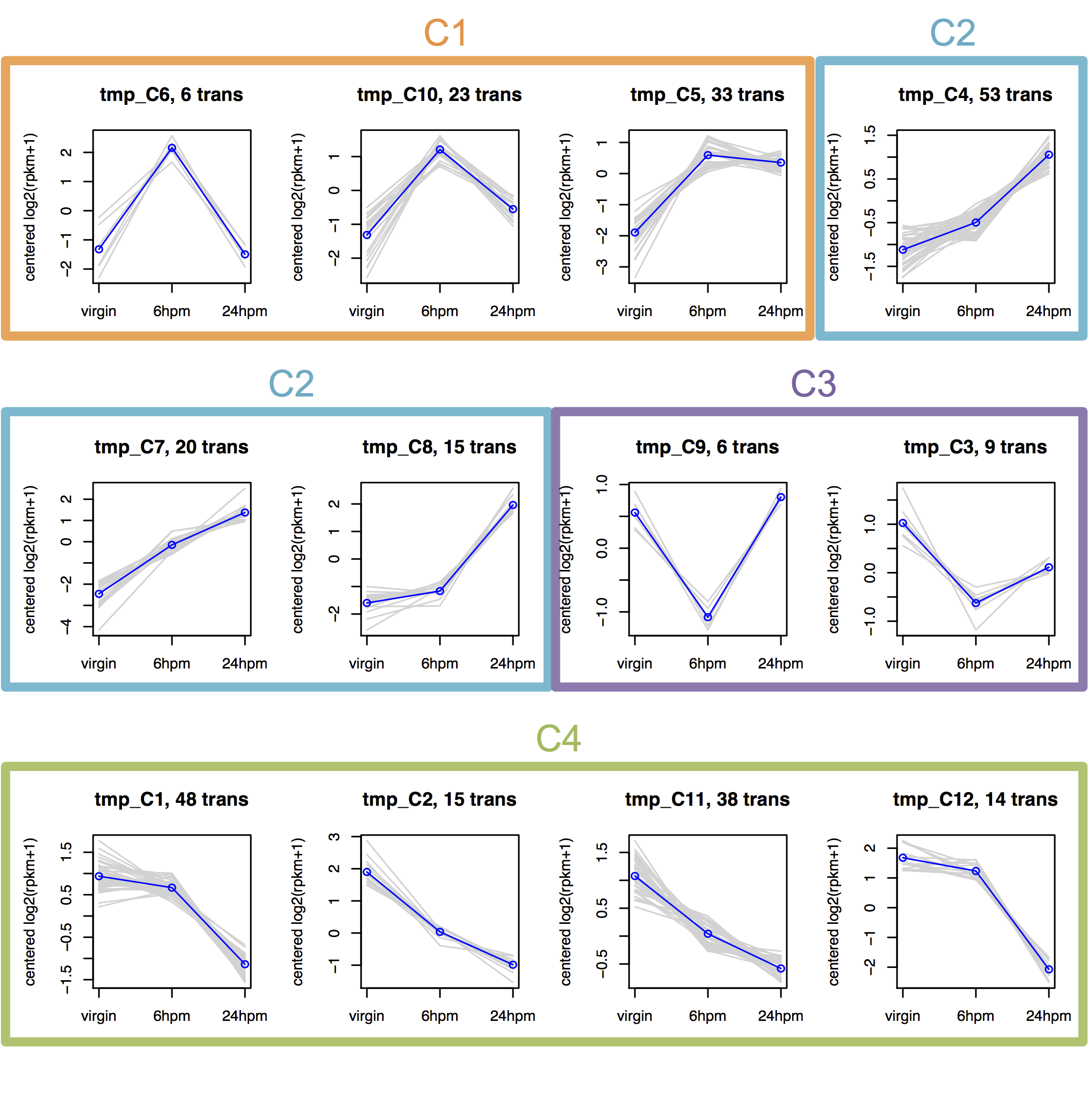

Supplement: S7 Fig — Initial K-means clusters for virgin versus 6hpm and 24hpm comparisons. Clusters were merged based on expression profile and time-point with maximum/minimum median abundance. (TIFF) [file pntd.0004451.s012.tiff]

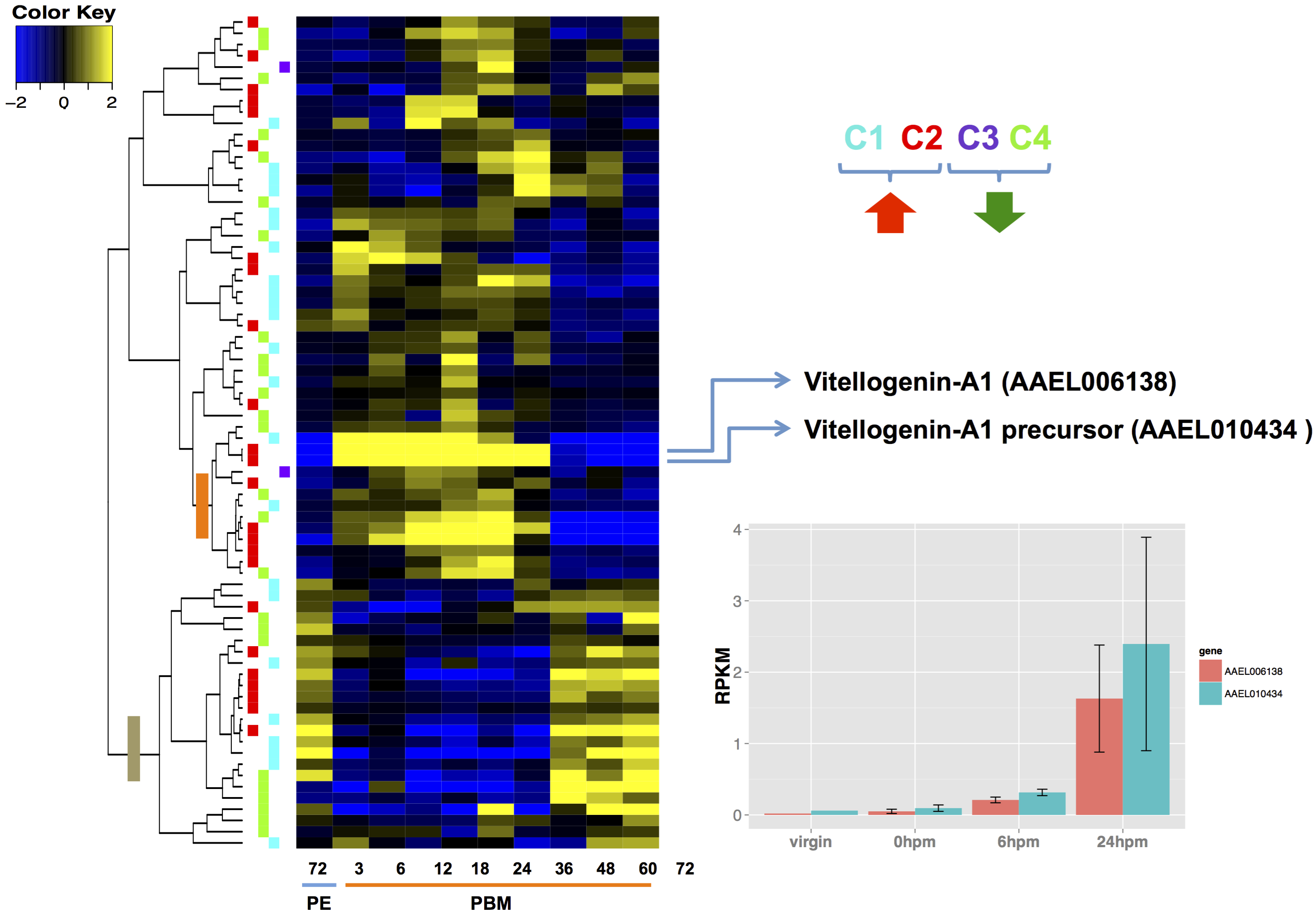

Supplement: S8 Fig — A comparison of expression patterns between our DE transcripts and those of Roy et al.[28] shows low level concordance among up- (orange bar on cladogram) and down-regulated (tan bar) transcripts in response to mating/blood feeding. Colored bars depicting merged K-means clusters from Fig 5A are indicated on the left of the heatmap. Two transcripts with the highest increase in abundance in response to mating/blood meal in the Roy et al. dataset are two vitellogenins, and those two are up-regulated in response to mating in our dataset, albeit at much lower levels. (TIFF) [file pntd.0004451.s013.tiff]

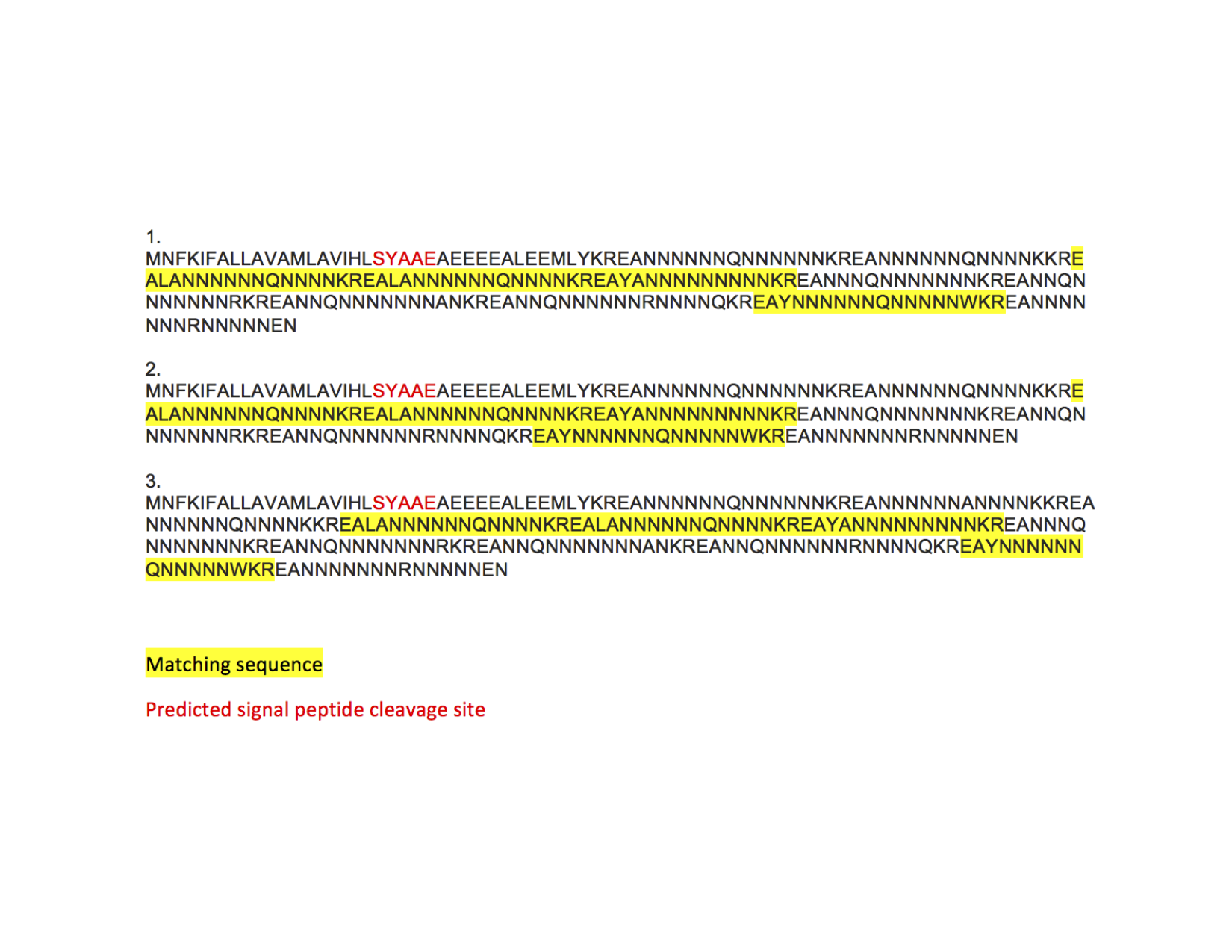

Supplement: S9 Fig — Amino acid sequences generated from XLOC019584 transcripts that exhibit sequence coverage in the mass spectrometry analysis of Ae. aegypti male accessory gland extract. (TIFF) [file pntd.0004451.s014.tiff]

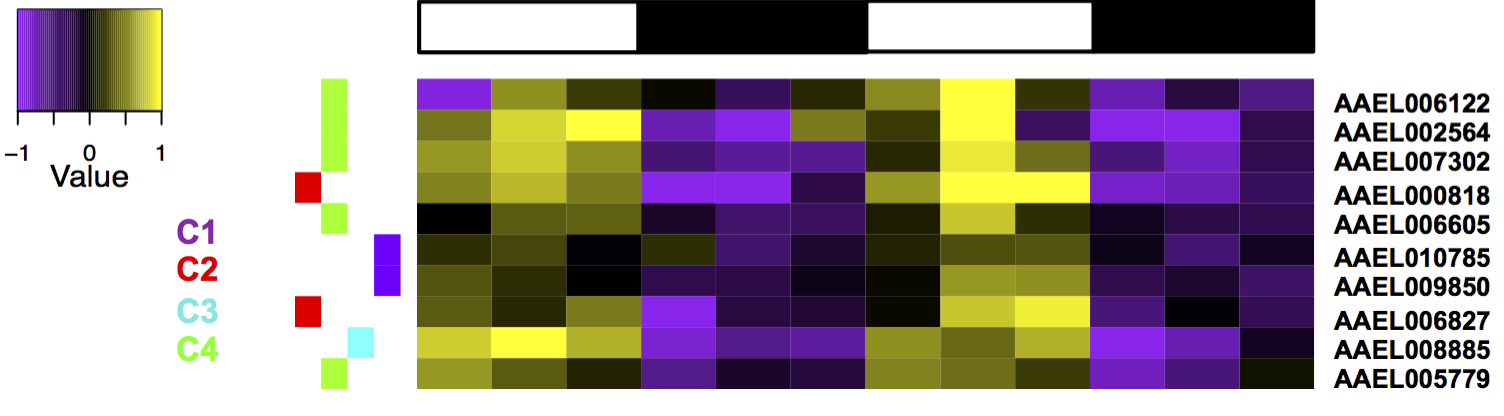

Supplement: S10 Fig — Ten of the 280 DE transcripts show cyclical expression pattern that corresponds to light/day changes. K-means cluster affiliation of each transcript is shown on the left. Light and Dark are indicated on top of the heatmap by white and black rectangles, respectively. (TIFF) [file pntd.0004451.s015.tiff]

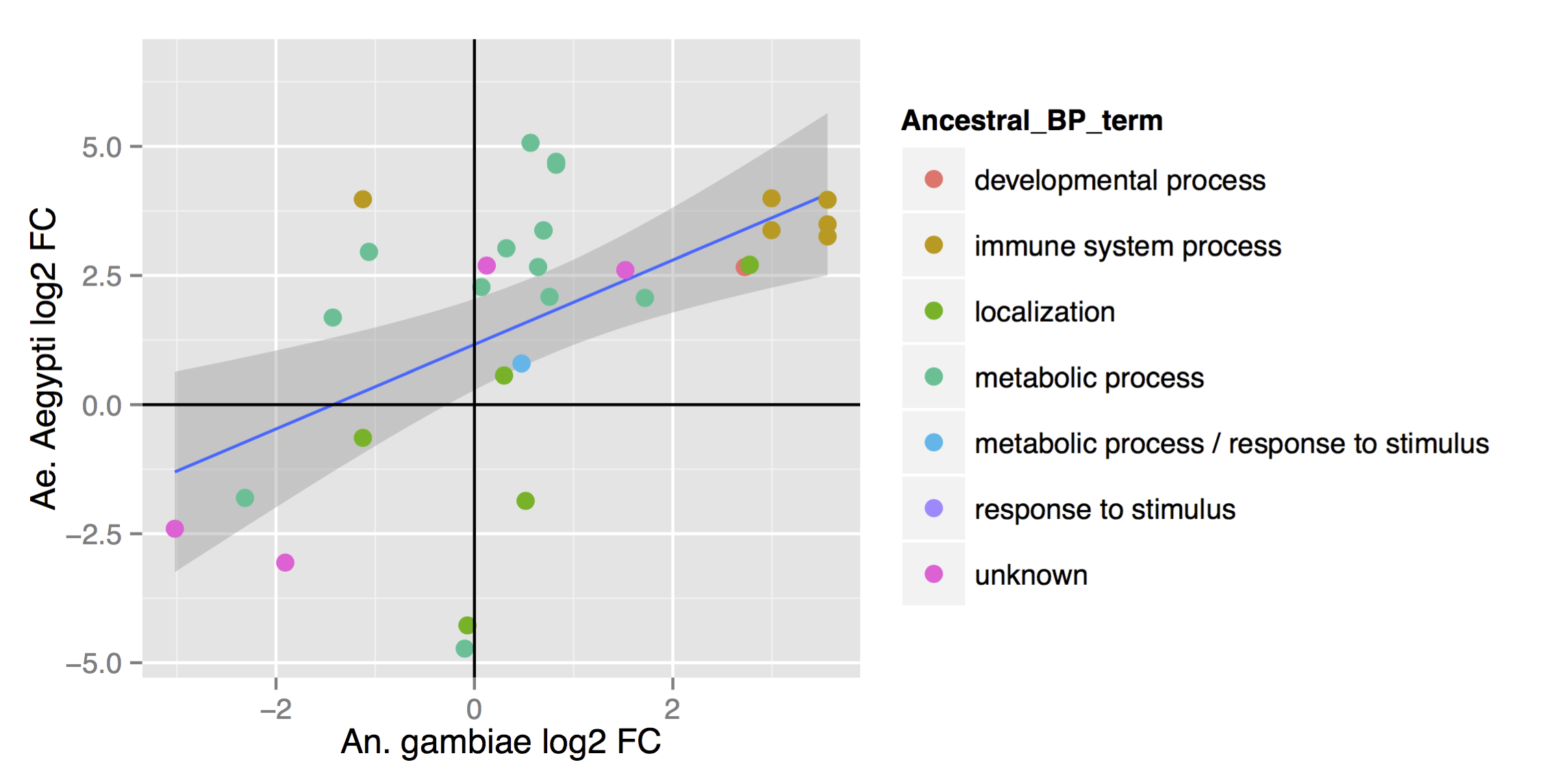

Supplement: S11 Fig — Fold-change estimates of DE transcripts in this study and their ortholgoues in An. gambiae[23] are shown and their ancestral Biological Process GO terms are indicated. A linear regression with 95% confidence interval is also shown. The data for this plot can be found in S4 Table. (TIFF) [file pntd.0004451.s016.tiff]
